# Synthesis of benzothiophene and indole derivatives through metal-free propargyl–allene rearrangement and allyl migration

**DOI:** 10.3762/bjoc.13.181

**Published:** 2017-09-06

**Authors:** Jinzhong Yao, Yajie Xie, Lianpeng Zhang, Yujin Li, Hongwei Zhou

**Affiliations:** 1 College of Biological, Chemical Sciences and Engineering, Jiaxing University, Jiaxing 314001, P. R. China

**Keywords:** allyl migration, benzothiophene, indole, metal-free, propargyl-allenyl

## Abstract

An efficient base-catalyzed protocol for the synthesis of benzothiophene is described. The reaction proceeds via base-promoted propargyl–allenyl rearrangement followed by cyclization and allyl migration. Phosphine-substituted indoles can be synthesized by a similar strategy.

## Introduction

Heterocycles are frequently found in natural products and pharmacologically active compounds, thus economic and efficient methods to construct heterocycles are always highly desirable [1–6].

**Figure 1:**
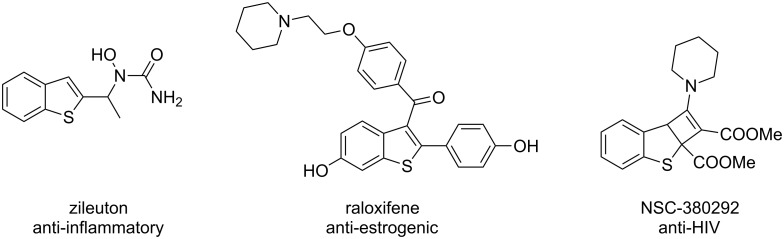
Examples of biologically active benzothiophene derivatives.

Benzothiophenes are important heterocycles that are one of the key motifs of anti-inflammatory, anti-estrogenic and anti-HIV drugs (Figure 1) [7–9]. Moreover, benzothiophenes have extensive applications in materials science. Besides the traditional methods of transition metal-catalyzed cyclization of alkyne substrates [10–12], the synthesis of benzothiophenes via metal-free conditions has recently aroused much attention [13–15]. For example, the preparation of C3-borylated benzothiophene by BCl_3_-induced borylative cyclization of arylalkynes was recently demonstrated by Ingleson [16].

**Scheme 1:**
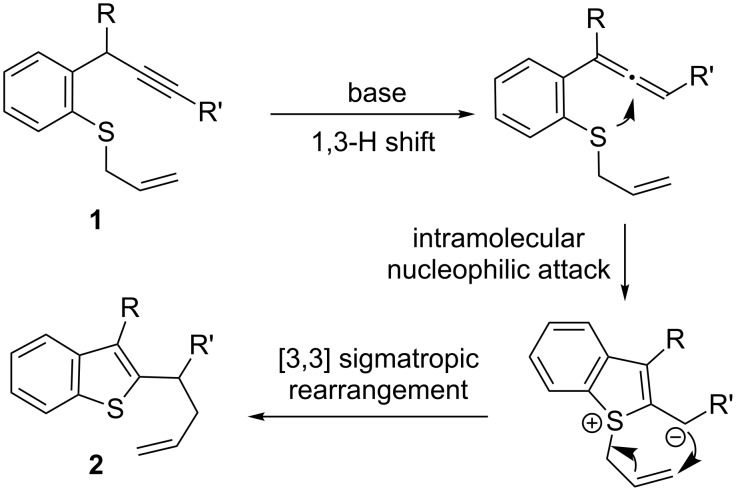
Proposal of applicable β-sulfonium carbanion.

Allene-mediated cyclization reactions are advantageous due to the convenient preparation of starting materials instead of the use of unstable or reactive polyfunctionalized allene substrates [17–27]. Although transition metal (e.g., Au, Pd)-catalysed propargyl–allenyl isomerization and cyclization reactions have been established [28–29], such transformations promoted by a base to construct heterocycles are not well-documented [30–31]. Recently, our group explored the utilization of β-sulfonium carbanions for the preparation of thiophene derivatives [19]. Alkynes were treated with acyl chloride under Sonogashira reaction conditions and the expected β-sulfonium carbanions were obtained in a one-pot process. Based on our understanding of organosulfur chemistry [20–22], we report herein a simple, metal-free method for the formation of benzothiophenes using an intramolecular addition of a sulfur atom (originated from a sulfide) to the electron-deficient allene moiety generated in situ by a propargyl–allenyl rearrangement [17–27] and an allyl migration [32–34] (Scheme 1). In addition, phosphine-substituted indole derivatives could also be conveniently constructed by a similar strategy. This method not only avoids the use of transition metal catalysts, but also provides the useful heterocycles which are not easily achieved through other protocols.

## Results and Discussion

In the initial studies, we treated methyl 4-(3-(2-(allylthio)phenyl)-3-methoxyprop-1-yn-1-yl)benzoate (**1a**) with DBU (0.1 equiv) in THF at 50 °C under N_2_ for 12 h (Table 1, entry 1). Fortunately, the desired product **2a** was obtained in 57% yield. No reaction was observed using TEA or DABCO, possibly because the allenic intermediate could not be formed by these comparatively weak bases (Table 1, entries 2 and 3), which was different from the previous work. Other bases, such as TBD, Cs_2_CO_3_, and *t*-BuOK were found to be less effective (Table 1, entries 4–6). To our delight, it was found that increasing the catalyst loading to 0.2 equiv resulted in an obviously higher yield of 83% (Table 1, entry 7). However, a higher catalyst loading had almost no influence on the reaction (Table 1, entry 8). It was found that THF was the best solvent after screening different solvents. Other solvents, such as DCE, toluene, and CH_3_CN were found to be less effective (Table 1, entries 9–11). The yield was reduced to 51% when the reaction time was decreased to 6 h (Table 1, entry 12). A lower temperature was found to be less effective for the reaction (Table 1, entry 13). Without the base, no reaction occurred, implying that the reaction proceeded exclusively through the allenic intermediate (Table 1, entry 14). Thus, the optimal reaction conditions were DBU (0.2 equiv) under nitrogen in THF at 50 °C for 12 h.

**Table 1:** Optimization of the reaction conditions^a^.

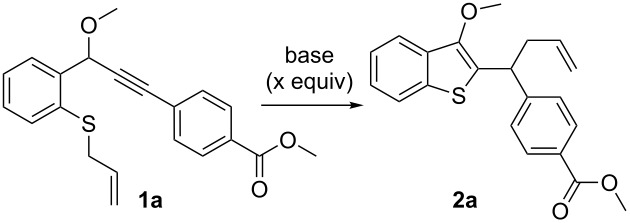				

Entry	Catalyst	x	Solvent	yield (%)^b^

1	DBU	0.1	THF	57
2	TEA	0.1	THF	N.D
3	DABCO	0.1	THF	N.D
4	TBD	0.1	THF	22
5	Cs_2_CO_3_	0.1	THF	23
6	*t*-BuOK	0.1	THF	27
7	DBU	0.2	THF	83
8	DBU	0.5	THF	82
9	DBU	0.2	DCE	62
10	DBU	0.2	toluene	68
11	DBU	0.2	CH_3_CN	58
12	DBU	0.2	THF	51^c^
13	DBU	0.2	THF	32^d^
14	–	–	THF	N.D

^a^Reaction conditions: **1a** (1.0 equiv), base (x equiv), 50 °C, 12 h, under N_2_. ^b^Isolated yield. ^c^The reaction time was 6 h. ^d^The reaction was conducted at 25 °C. DBU: 1,8-diazabicyclo[5.4.0]undec-7-ene. TBD: 1,5,7-triazabicyclo[4.4.0]dec-5-ene.

**Figure 2:**
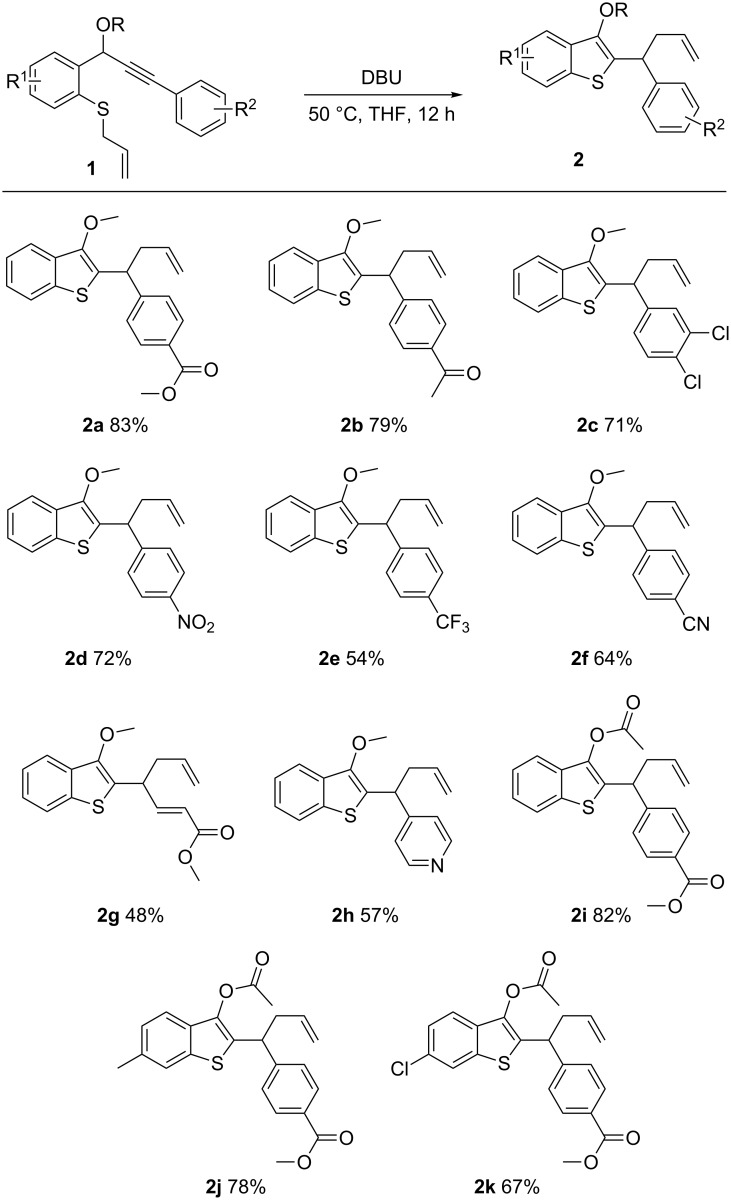
Synthesis of benzothiophenes. Reaction conditions: **1** (0.5 mmol), DBU (0.1 mmol), THF (2.0 mL), 50 °C, 12 h, under N_2_. Yields are isolated yields.

With the optimized reaction conditions in hand, we turned our attention to study the reaction scope and limitations of this reaction; the results are shown in Figure 2. A series of alkynes substituted with an electron-withdrawing group participated in this reaction smoothly to give the products in good yields (**2a**–**k**). A variety of substituents, such as *p*-COOEt, *p*-COCH_3_, dichloro, *p*-NO_2_, *p*-CF_3_ and *p*-CN were well-tolerated during the reaction, leading to **2a**–**f** in 54–83% yield. The presence of methyl acrylate or pyridine was also well-tolerated, as exemplified in the formation of **2g**,**h** in 48–57% yield. Besides methyl propargyl ethers, propargyl acetates were also tolerated under these conditions (**2i**–**k**). The presence of substituents on the aromatic ring, such as a methyl group or a chlorine atom, did not have much of an effect the reaction (**2j**,**k**).

**Scheme 2:**
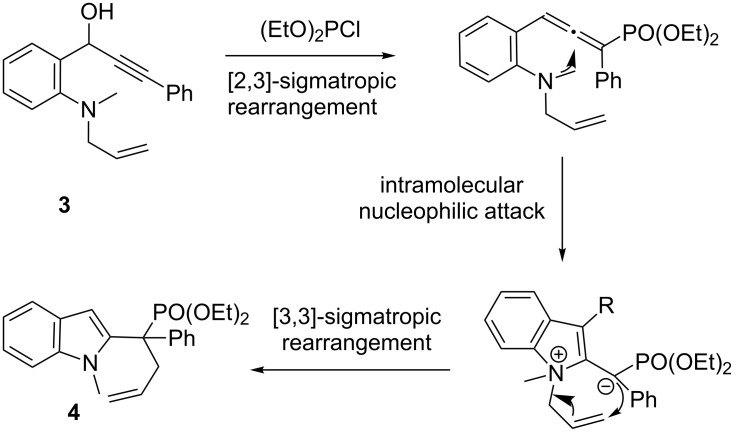
Proposal of indole synthesis via allenylphosphonates.

**Figure 3:**
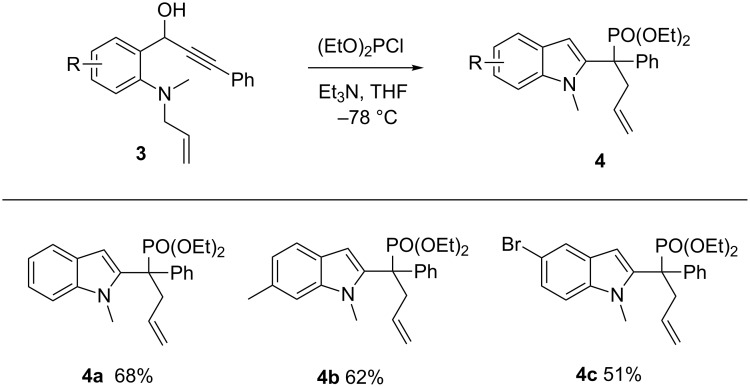
Synthesis of 1-methylindole phosphine oxides. Reaction conditions: **3** (0.5 mmol), (EtO)_2_PCl (0.6 mmol), Et_3_N (1.5 mmol), and THF (2.0 mL) at −78 °C. Yields are isolated yield.

Indoles are also important heterocycles that are the key motif of many natural products and pharmaceuticals. Consequently, new and straightforward methods to access indoles are highly desirable [35–36]. We chose a propargyl phosphite rearrangement to achieve allenyl intermediates and aimed to synthetize indoles via allenyl phosphonates, which were versatile synthetic intermediates [37–38]. The *N*-methyl-*N*-allylpropargyl alcohol **3** was treated with (EtO)_2_PCl under alkaline conditions, then underwent a propargyl phosphite/allenyl phosphonate rearrangement and an intramolecular nucleophilic attack to form the indole moiety, followed by allyl migration (Scheme 2). Phosphine-substituted indole derivatives were obtained in moderate yield (Figure 3, **4a**–**c**).

## Conclusion

In summary, we have developed an expedient route for the construction of benzothiophene and indole derivatives via propargyl–allene rearrangement and allyl migration. The reaction proceeded under mild conditions to produce useful benzothiophene and indole derivatives.

## Supporting Information

File 1 Experimental procedures and analytical data.
