# Supplementary material for: Synthesis of benzothiophene and indole derivatives through metal-free propargyl–allene rearrangement and allyl migration
Source: Beilstein J Org Chem. 2017 Sep 6;13:1866–70. doi: 10.3762/bjoc.13.181 (PMC5629416; doi:10.3762/bjoc.13.181)
Supplement: File 1 — Experimental procedures and analytical data. [file Beilstein_J_Org_Chem-13-1866-s001.pdf]

Supporting Information

for

Synthesis of benzothiophene and indole derivatives through metal-free propargyl–allene rearrangement and allyl migration

Jinzhong Yao*, Yajie Xie, Lianpeng Zhang, Yujin Li and Hongwei Zhou*

Address: College of Biological, Chemical Sciences and Engineering, Jiaxing University, Jiaxing 314001, P. R. China

Email: Jinzhong Yao* - jzyao@zju.edu.cn; Hongwei Zhou* - zhouhw@zju.edu.cn

* Corresponding author

Experimental procedures and analytical data

Table of contents

General information S2

Experimental procedure S2

Characterization data of the products S3

The ^{1}H and ^{13}C NMR spectra of compounds S9

1. General information

Tetrahydrofuran was dried with Na and distilled freshly before use. Et_3N was dried with NaH and distilled freshly before use. Other materials and solvents were purchased from commercial suppliers and used without additional purification. NMR spectra were measured in $CDCl_3$ and recorded on Bruker Avance spectrometers operating for ^{1}H NMR at 400 MHz, for ^{13}C NMR at 100 MHz and for ^{31}P at 160 MHz. Chemical shifts are expressed in ppm and *J* values are given in Hz. Mass spectrometry data of the products were collected with an HRMS–TOF instrument GCT Premier, which is produced by WATERS company, and the collision energy is 70 eV. Infrared spectra were recorded with a Bruker ATRFTIR spectrometer.

2. Experimental procedures:

Optimization of the reaction conditions:

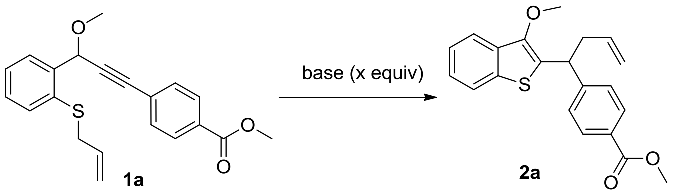

Entry	Catalyst	x	Solvent	yield (%)[b]
1	DBU	0.1	THF	57
2	TEA	0.1	THF	N.D
3	DABCO	0.1	THF	N.D
4	TBD	0.1	THF	22
5	Cs_2CO_3	0.1	THF	23
6	*t*-BuOK	0.1	THF	27
7	DBU	0.2	THF	83
8	DBU	0.5	THF	82
9	DBU	0.2	DCE	62

10	DBU	0.2	toluene	68
11	DBU	0.2	CH_3CN	58
12	DBU	0.2	THF	51[c]
13	DBU	0.2	THF	32[d]
14	/	/	THF	N.D

[a]Reaction conditions: **1a** (1.0 equiv), base (x equiv), 50 °C, 12 h, under N_2. [b]Isolated yield. [c]The reaction time was 6 h. [d]The reaction was conducted at 25 °C. DBU was short for 1,8-diazabicyclo[5.4.0]undec-7-ene. TBD was short for 1,5,7-triazabicyclo[4.4.0]dec-5-ene.

Typical procedure for the synthesis of 2a–k: To a 25 mL Schlenk tube with **1** (0.5 mmol) was added DBU (0.1 mmol) in tetrahydrofuran (2.0 mL) under N_2 atmosphere. The mixture was stirred at 50 ^{o}C for 12 h. Then the reaction was quenched with water (10 mL), extracted with ethyl acetate (3 × 10 mL), dried with anhydrous Na_2SO_4. After evaporation, chromatography on silica gel (ethyl acetate / petroleum ether = 1:10, v/v) of the reaction mixture afforded the desired product.

Typical procedure for the synthesis of 4a–c: To a solution of alcohol **3** (0.5 mmol) in tetrahydrofuran (2.0 mL) was added triethylamine (1.5 mmol) under N_2 atmosphere at −78 °C. The resulting mixture was stirred at −78 °C for 10 minutes. Then diethyl phosphorochloridite (0.6 mmol) was added at −78 °C and the reaction was monitored by TLC until completion. Then the reaction was quenched with water (15 mL), extracted with ethyl acetate (3 × 10 mL), dried with anhydrous Na_2SO_4. After evaporation, chromatography on silica gel (ethyl acetate / petroleum ether = 1:2, v/v) of the reaction mixture afforded the desired product.

3. Characterization data of the products:

Methyl 4-(1-(3-methoxybenzo[*b*]thiophen-2-yl)but-3-en-1-yl)benzoate (2a)

Yellow oil; 146 mg, 83% yield; ^{1}H NMR (400 MHz, $CDCl_3$, TMS) δ 8.01 (d, J = 8.0 Hz, 2H), 7.71 (t, J = 8.0 Hz, 2H), 7.49 (d, J = 8.0 Hz, 2H), 7.37-7.25 (m, 2H),

5.83-5.72 (m, 1H), 5.12 (d, *J* = 16 Hz, 1H), 5.02 (d, *J* = 12 Hz, 1H), 4.66 (t, *J* = 8.0 Hz, 1H), 3.90 (s, 3H), 3.84 (s, 3H), 2.90-2.86 (m, 2H); ^{13}C NMR (100 MHz, $CDCl_3$) δ 166.9, 148.7, 147.4, 136.1, 135.6, 133.2, 131.9, 129.9, 128.5, 127.8, 124.4, 124.1, 123.1, 120.6, 117.4, 61.6, 52.1, 43.1, 40.4; IR (neat) 2943, 1721, 734; HRMS (EI) Calcd for $C_{21}H_{20}O_3S$ $(M)^+$ 352.1133; Found, 352.1121.

1-(4-(1-(3-Methoxybenzo[*b*]thiophen-2-yl)but-3-en-1-yl)phenyl)ethanone (2b)

Yellow oil; 133 mg, 79% yield; ^{1}H NMR (400 MHz, $CDCl_3$, TMS) δ 7.89 (d, *J* = 8.0 Hz, 2H), 7.70-7.66 (m, 2H), 7.48 (d, *J* = 8.0 Hz, 2H), 7.34-7.24 (m, 2H), 5.80-5.69 (m, 1H), 5.09 (d, *J* = 16 Hz, 1H), 4.99 (d, *J* = 8.0 Hz, 1H), 4.63 (t, *J* = 8.0 Hz, 1H), 3.83(s, 3H), 2.87-2.83 (m, 2H), 2.55 (s, 3H); ^{13}C NMR (100 MHz, $CDCl_3$) δ 197.6, 148.9, 147.4, 136.1, 135.6, 135.5, 133.2, 131.8, 128.7, 127.9, 124.4, 124.1, 123.1, 120.6, 117.4, 61.6, 43.1, 40.3, 26.6; IR (neat) 2928, 1680, 615; HRMS (EI) Calcd for $C_{21}H_{20}O_2S$ $(M)^+$ 336.1184; Found, 336.1182.

2-(1-(3,4-Dichlorophenyl)but-3-en-1-yl)-3-methoxybenzo[*b*]thiophene (2c)

Yellow oil; 129 mg, 71% yield; ^{1}H NMR (400 MHz, $CDCl_3$, TMS) δ 7.72 (t, *J* = 8.0 Hz, 2H), 7.49 (d, *J* = 4.0 Hz, 1H), 7.39-7.29 (m, 3H), 7.25-7.22 (m, 1H), 5.80-5.70 (m, 1H), 5.12 (d, *J* = 16 Hz, 1H), 5.03 (d, *J* = 8 Hz, 1H), 4.55 (t, *J* = 8.0 Hz, 1H), 3.87 (s, 3H), 2.84-2.82 (m, 2H); ^{13}C NMR (100 MHz, $CDCl_3$) δ 147.4, 143.8, 136.1, 135.3, 133.2, 132.5, 131.5, 130.6, 130.4, 129.7, 127.2, 124.5, 124.2, 123.1, 120.7, 117.6, 61.7, 42.3, 40.3; IR (neat) 2926, 1466, 1192; HRMS (EI) Calcd for $C_{19}H_{16}Cl_2OS$ $(M)^+$ 362.0299; Found, 362.0301.

3-Methoxy-2-(1-(4-nitrophenyl)but-3-en-1-yl)benzo[*b*]thiophene (2d)

Yellow oil; 122 mg, 72% yield; ^{1}H NMR (400 MHz, $CDCl_3$, TMS) δ 8.17 (d, *J* = 12 Hz, 2H), 7.71 (dd, *J* = 12, 8.0 Hz, 2H), 7.55 (d, *J* = 8.0 Hz, 2H), 7.38-7.26 (m, 2H), 5.79-5.73 (m, 1H), 5.12 (d, *J* = 16 Hz, 1H), 5.03 (d, *J* = 8.0 Hz, 1H), 4.69 (t, *J* = 8.0 Hz, 1H), 3.86 (s, 3H), 2.91-2.85 (m, 2H); ^{13}C NMR (100 MHz, $CDCl_3$) δ 151.0, 147.7, 146.6, 136.1, 135.0, 133.1, 130.8, 128.6, 124.6, 124.3, 123.8, 123.1, 120.7, 117.9, 61.7, 42.9, 40.2; IR (neat) 3414, 1616, 1190; HRMS (EI) Calcd for $C_{19}H_{17}NO_3S$ $(M+Na)^+$ 362.0821; Found, 362.0817.

3-Methoxy-2-(1-(4-(trifluoromethyl)phenyl)but-3-en-1-yl)benzo[*b*]thiophene (2e)

Yellow oil; 97.7 mg, 54% yield; ^{1}H NMR (400 MHz, $CDCl_3$, TMS) δ 7.70 (dd, J = 12, 8.0 Hz, 2H), 7.56 (d, J = 12 Hz, 2H), 7.50 (d, J = 8.0 Hz, 2H), 7.36-7.25 (m, 2H), 5.80-5.70 (m, 1H), 5.11 (d, J = 16 Hz, 1H), 5.01 (d, J = 12 Hz, 1H), 4.64 (t, J = 8.0 Hz, 1H), 3.84 (s, 3H), 2.88-2.83 (m, 2H); ^{13}C NMR (100 MHz, $CDCl_3$) δ 147.5, 147.4, 136.1, 135.4, 133.2, 131.7, 128.9 (q, J = 32.7 Hz), 128.07, 125.5 (q, J = 3.7 Hz), 124.5, 124.2, 124.2 (q, J = 271 Hz), 123.1, 120.6, 117.5, 61.7, 42.9, 40.4; IR (neat) 2921, 1385, 616; HRMS (EI) Calcd for $C_{20}H_{17}F_3OS$ $(M+Na)^+$ 385.0850; Found, 385.0862.

4-(1-(3-Methoxybenzo[*b*]thiophen-2-yl)but-3-en-1-yl)benzonitrile (2f)

Yellow oil; 102 mg, 64% yield; ^{1}H NMR (400 MHz, $CDCl_3$, TMS) δ 7.70 (t, J = 8.0 Hz, 2H), 7.60 (d, J = 8.0 Hz, 2H), 7.50 (d, J = 4.0 Hz, 2H), 7.37-7.25 (m, 2H), 5.77-5.69 (m, 1H), 5.11 (d, J = 16 Hz, 1H), 5.02 (d, J = 8.0 Hz, 1H), 4.63 (t, J = 8.0 Hz, 1H), 3.84 (s, 3H), 2.88-2.82 (m, 2H); ^{13}C NMR (100 MHz, $CDCl_3$) δ 148.9, 147.6, 136.1, 135.1, 133.1, 132.4, 131.0, 128.6, 124.6, 124.2, 123.1, 120.7, 118.9, 117.7, 110.5, 61.7, 43.1, 40.1; IR (neat) 3068, 2228, 735; HRMS (EI) Calcd for $C_{20}H_{17}NOS$ $(M+Na)^+$ 342.0929; Found, 342.0926.

Methyl 4-(3-methoxybenzo[*b*]thiophen-2-yl)hepta-2,6-dienoate (2g)

Yellow oil; 72.5 mg, 48% yield; ^{1}H NMR (400 MHz, $CDCl_3$, TMS) δ 7.69 (dd, J = 12, 8.0 Hz, 2H), 7.35-7.24 (m, 2H), 6.35 (t, J = 8.0 Hz, 1H), 5.84 (d, J = 12 Hz, 1H), 5.81-5.72 (m, 1H), 5.41 (dd, J = 20, 8.0 Hz, 1H), 5.08 (d, J = 20 Hz, 1H), 5.00 (d, J = 8.0 Hz, 1H), 3.92 (s, 3H), 3.74 (s, 3H), 2.61-2.48 (m, 2H); ^{13}C NMR (100 MHz, $CDCl_3$) δ 166.3, 150.0, 147.7, 135.9, 135.0, 133.5, 130.3, 124.3, 124.0, 123.0, 120.5, 119.0, 117.2, 61.7, 51.3, 40.7, 36.3; IR (neat) 3415, 1720, 619; HRMS (EI) Calcd for $C_{17}H_{18}O_3S$ $(M)^+$ 302.0977; Found, 302.0979.

4-(1-(3-Methoxybenzo[*b*]thiophen-2-yl)but-3-en-1-yl)pyridine (2h)

Yellow oil; 84 mg, 57% yield; ^{1}H NMR (400 MHz, $CDCl_3$, TMS) δ 8.52 (s, 2H), 7.69 (dd, J = 12, 8.0 Hz, 2H), 7.37-7.24 (m, 4H), 5.77-5.67 (m, 1H), 5.09 (d, J = 16 Hz, 1H), 5.01 (d, J = 12 Hz, 1H), 4.57 (t, J = 8.0 Hz, 1H), 3.83 (s, 3H), 2.82 (t, J = 8.0 Hz,

2H); ^{13}C NMR (100 MHz, $CDCl_3$) δ 152.7, 149.5, 147.8, 136.1, 135.0, 133.0, 130.4, 124.6, 124.2, 123.1, 120.7, 117.8, 61.6, 42.4, 39.8; IR (neat) 3414, 1619, 618; HRMS (EI) Calcd for $C_{18}H_{17}NOS$ $(M)^+$ 295.1031; Found, 295.1038.

Methyl 4-(1-(3-acetoxybenzo[*b*]thiophen-2-yl)but-3-en-1-yl)benzoate (2i)

Yellow oil; 156 mg, 82% yield; ^{1}H NMR (400 MHz, $CDCl_3$, TMS) δ 7.99 (d, J = 12 Hz, 2H), 7.71 (d, J = 8.0 Hz, 1H), 7.40-7.38 (m, 3H), 7.35-7.26 (m, 2H), 5.78-5.68 (m, 1H), 5.09 (d, J = 20 Hz, 1H), 5.01 (d, J = 8.0 Hz, 1H), 4.39 (t, J = 8.0 Hz, 1H), 3.89 (s, 3H), 2.94-2.81 (m, 2H), 2.33 (s, 3H); ^{13}C NMR (100 MHz, $CDCl_3$) δ 168.4, 166.8, 147.4, 137.2, 135.6, 135.1, 134.1, 132.7, 129.9, 128.8, 127.8, 124.8, 124.5, 122.8, 120.2, 117.6, 52.1, 43.6, 39.9, 20.5; IR (neat) 3414, 1720, 733; HRMS (EI) Calcd for $C_{22}H_{20}O_4S$ $(M+Na)^+$ 403.0980; Found, 403.0975.

Methyl 4-(1-(3-acetoxy-6-methylbenzo[*b*]thiophen-2-yl)but-3-en-1-yl)benzoate (2j)

Yellow oil; 154 mg, 78% yield; ^{1}H NMR (400 MHz, $CDCl_3$, TMS) δ 7.98 (d, J = 8.0 Hz, 2H), 7.50 (s, 1H), 7.38 (d, J = 8.0 Hz, 2H), 7.29-7.26 (m, 1H), 7.15 (d, J = 8.0 Hz, 1H), 5.77-5.67 (m, 1H), 5.08 (d, J = 16 Hz, 1H), 5.01 (d, J = 12 Hz, 1H), 4.36 (t, J = 8.0 Hz, 1H), 3.89 (s, 3H), 2.93-2.81 (m, 2H), 2.43 (s, 3H), 2.31 (s, 3H); ^{13}C NMR (100 MHz, $CDCl_3$) δ 168.4, 166.9, 147.6, 137.2, 135.9, 135.2, 134.8, 132.6, 130.5, 129.9, 128.7, 127.8, 126.2, 122.6, 119.9, 117.5, 52.1, 43.5, 39.9, 21.6, 20.5; IR (neat) 3414, 1719, 618; HRMS (EI) Calcd for $C_{23}H_{22}O_4S$ $(M)^+$ 417.1136; Found, 417.1140.

Methyl 4-(1-(3-acetoxy-6-chlorobenzo[*b*]thiophen-2-yl)but-3-en-1-yl)benzoate (2k)

Yellow oil; 139 mg, 67% yield; ^{1}H NMR (400 MHz, $CDCl_3$, TMS) δ 7.98 (d, J = 8.0 Hz, 2H), 7.69 (s, 1H), 7.36 (d, J = 8.0 Hz, 2H), 7.29 (s, 2H), 5.75-5.65 (m, 1H), 5.08 (d, J = 16 Hz, 1H), 5.01 (d, J = 8.0 Hz, 1H), 4.35 (t, J = 8.0 Hz, 1H), 3.89 (s, 3H), 2.88-2.82 (m, 2H), 2.31 (s, 3H); ^{13}C NMR (100 MHz, $CDCl_3$) δ 168.3, 166.8, 147.1, 136.9, 136.5, 134.8, 134.7, 131.2, 130.8, 120.0, 128.9, 127.8, 125.4, 122.4, 121.2, 117.8, 52.1, 43.6, 39.8, 20.5; IR (neat) 3414, 1774, 616; HRMS (EI) Calcd for $C_{22}H_{19}ClO_4S$ $(M)^+$ 414.0693; Found, 414.0698.

Diethyl (1-(1-methyl-1*H*-indol-2-yl)-1-phenylbut-3-en-1-yl)phosphonate (4a)

Yellow oil; 135 mg, 68% yield; ^{1}H NMR (400 MHz, $CDCl_3$, TMS) δ 7.60 (d, J = 8.0 Hz, 1H), 7.49 (d, J = 8.0 Hz, 2H), 7.31–7.26 (m, 2H), 7.23–7.18 (m, 3H), 7.12–7.08 (m, 1H), 5.94 (s, 1H), 5.15–5.04 (m, 2H), 5.01 (d, J = 8.0 Hz, 1H), 4.23-4.13 (m, 2H), 3.92-3.86 (m, 1H), 3.64-3.61 (m, 2H), 3.56 (s, 3H), 3.54-3.47 (m, 1H), 1.34 (t, J = 8.0 Hz, 3H), 1.04 (t, J = 8.0 Hz, 3H); ^{13}C NMR (100 MHz, $CDCl_3$) δ 137.8, 137.2 (d, J = 3.0 Hz), 135.3, 131.0 (d, J = 9.0 Hz), 129.1, 129.0, 128.5, 127.4 (d, J = 2.0 Hz), 127.0, 121.6, 119.0, 115.0, 112.4 (d, J = 9.0 Hz), 109.1, 63.1 (d, J = 7.0 Hz), 62.1 (d, J = 8.0 Hz), 41.2 (d, J = 143 Hz), 30.6 (d, J = 170 Hz), 29.2, 16.4 (d, J = 5.0 Hz), 16.3 (d, J = 5.0 Hz); ^{31}P NMR (160 MHz, $CDCl_3$) δ 23.5; IR (neat) 3415, 1620, 697; HRMS (EI) Calcd for $C_{23}H_{28}NO_3P$ $(M+Na)^+$ 420.1704; Found, 420.1701.

Diethyl (1-(1,6-dimethyl-1*H*-indol-2-yl)-1-phenylbut-3-en-1-yl)phosphonate (4b)

Yellow oil; 127 mg, 62% yield; ^{1}H NMR (400 MHz, $CDCl_3$, TMS) δ 7.49-7.47 (m, 3H), 7.30-7.21 (m, 3H), 7.04 (s, 1H), 6.94 (d, J = 8.0 Hz, 1H), 5.96 (s, 1H), 5.12 (d, J = 16 Hz, 1H), 5.08–4.99 (m, 2H), 4.23-4.13 (m, 2H), 3.92-3.86 (m, 1H), 3.60 (s, 2H), 3.52 (s, 3H), 3.49-3.46 (m, 1H), 2.48 (s, 3H), 1.34 (t, J = 8.0 Hz, 3H), 1.04 (t, J = 8.0 Hz, 3H); ^{13}C NMR (100 MHz, $CDCl_3$) δ 137.8, 137.3 (d, J = 2.0 Hz), 135.4, 131.5, 130.3 (d, J = 8.0 Hz), 129.1, 129.0, 128.5, 126.9, 120.7, 118.7, 114.9, 112.3 (d, J = 2.0 Hz), 109.2, 63.1 (d, J = 7.0 Hz), 62.0 (d, J = 8.0 Hz), 41.6 (d, J = 137 Hz), 30.0 (d, J = 164 Hz), 29.3, 21.9, 16.4 (d, J = 6.0 Hz), 16.3 (d, J = 6.0 Hz); ^{31}P NMR (160 MHz, $CDCl_3$) δ 23.6; IR (neat) 3415, 1620, 618; HRMS (EI) Calcd for $C_{24}H_{30}NO_3P$ $(M)^+$ 434.1861; Found, 434.1860.

Diethyl (1-(5-bromo-1-methyl-1*H*-indol-2-yl)-1-phenylbut-3-en-1-yl)phosphonate (4c)

Yellow oil; 121 mg, 51% yield; ^{1}H NMR (400 MHz, $CDCl_3$, TMS) δ 7.69 (s, 1H), 7.46 (d, J = 4.0 Hz, 2H), 7.31–7.27 (m, 3H), 7.26–7.22 (m, 1H), 7.09 (d, J = 8.0 Hz, 1H), 5.91 (s, 1H), 5.11 (d, J = 16 Hz, 1H), 5.07–5.00 (m, 2H), 4.22-4.15 (m, 2H), 3.91-3.85 (m, 1H), 3.57-3.56 (m, 2H), 3.55 (s, 3H), 3.51-3.48 (m, 1H), 1.33 (t, J = 8.0 Hz, 3H), 1.03 (t, J = 8.0 Hz, 3H); ^{13}C NMR (100 MHz, $CDCl_3$) δ 136.4 (d, J = 69 Hz),

134.9, 132.4 (d, J = 9.0 Hz), 129.1, 129.0, 128.6, 127.1, 124.4, 121.4, 115.4, 111.4, 112.0 (d, J = 8.0 Hz), 110.7, 105.0, 63.2 (d, J = 7.0 Hz), 62.2 (d, J = 8.0 Hz), 41.7 (d, J = 143 Hz), 31.2 (d, J = 190 Hz), 29.0, 16.4 (d, J = 6.0 Hz), 16.3 (d, J = 6.0 Hz); ^{31}P NMR (160 MHz, $CDCl_3$) δ 23.1; IR (neat) 3416, 1621, 698; HRMS (EI) Calcd for $C_{23}H_{27}BrNO_3P$ $(M)^+$ 498.0810; Found, 498.0807.

4. The ^{1}H and ^{13}C NMR spectra of compounds

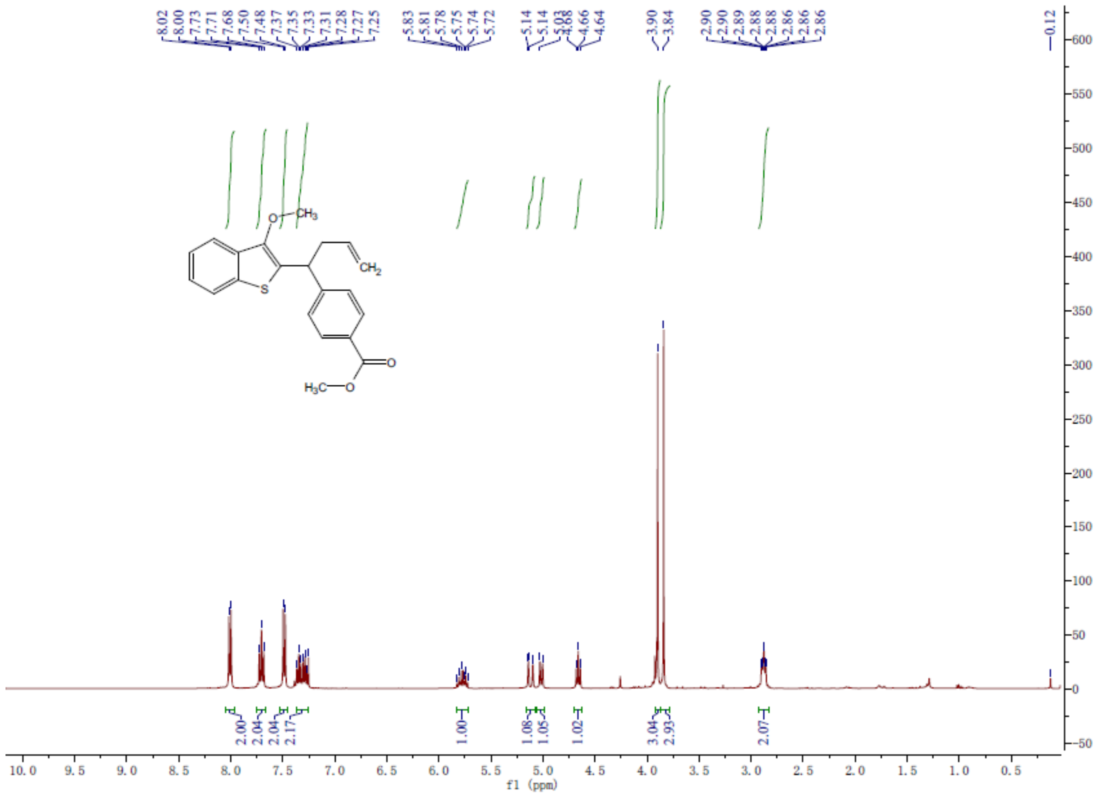

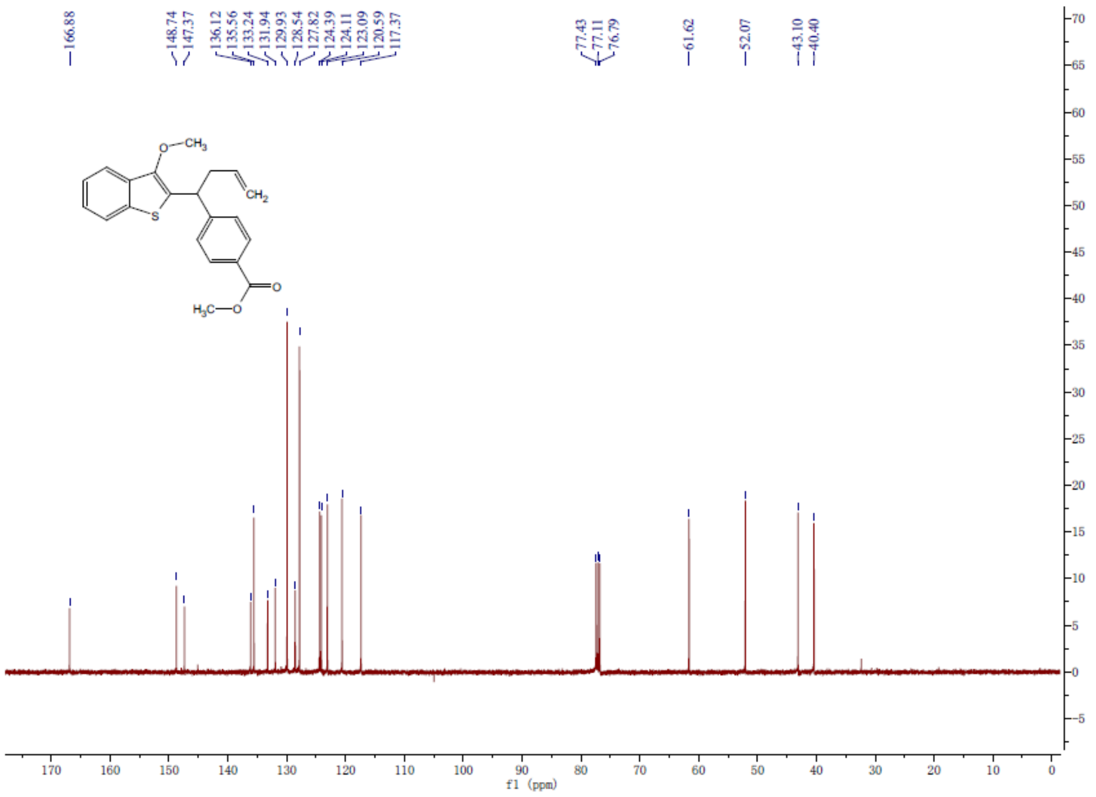

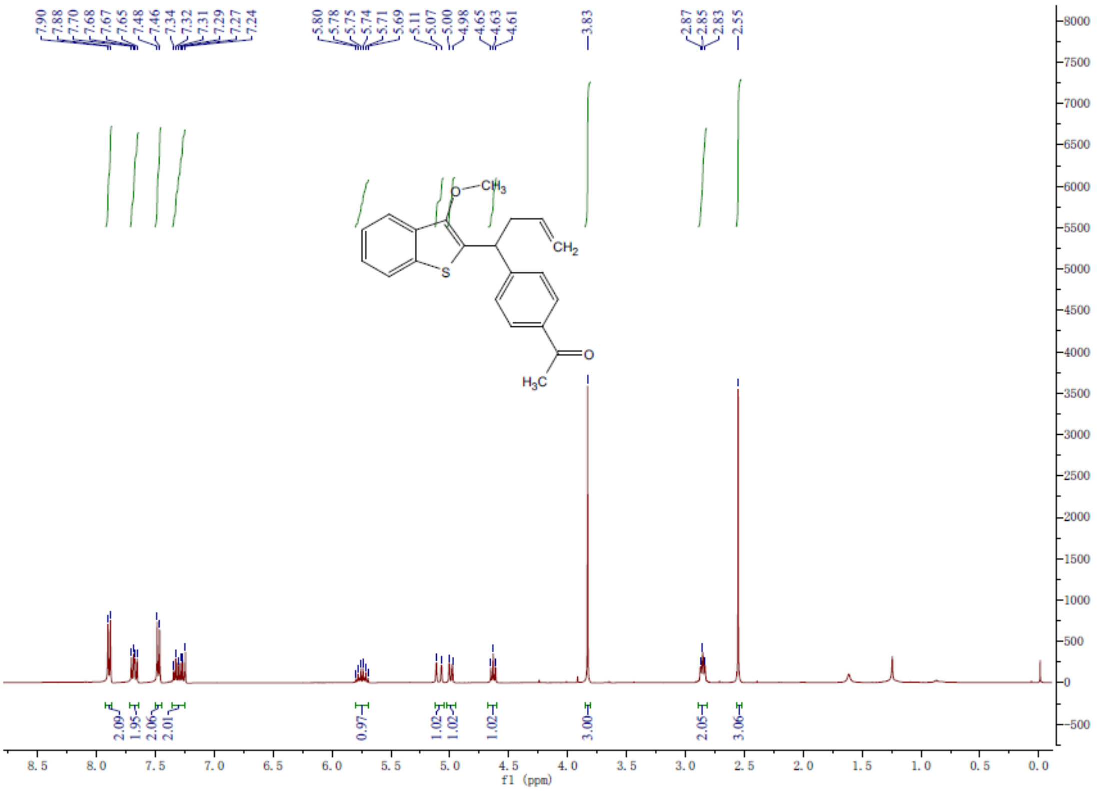
7.90 7.88 7.70 7.68 7.67 7.65 7.48 7.46 7.34 7.32 7.31 7.29 7.27 7.24
5.80 5.78 5.75 5.74 5.71 5.69 5.11 5.07 5.00 4.98 4.65 4.63 4.61
3.83
2.87 2.85 2.83 2.55
O
CH3
CH2
S
O
H3C
2.09 1.95 2.06 2.01 0.97 1.02 1.02 1.02 3.00 2.05 3.06
f1 (ppm)

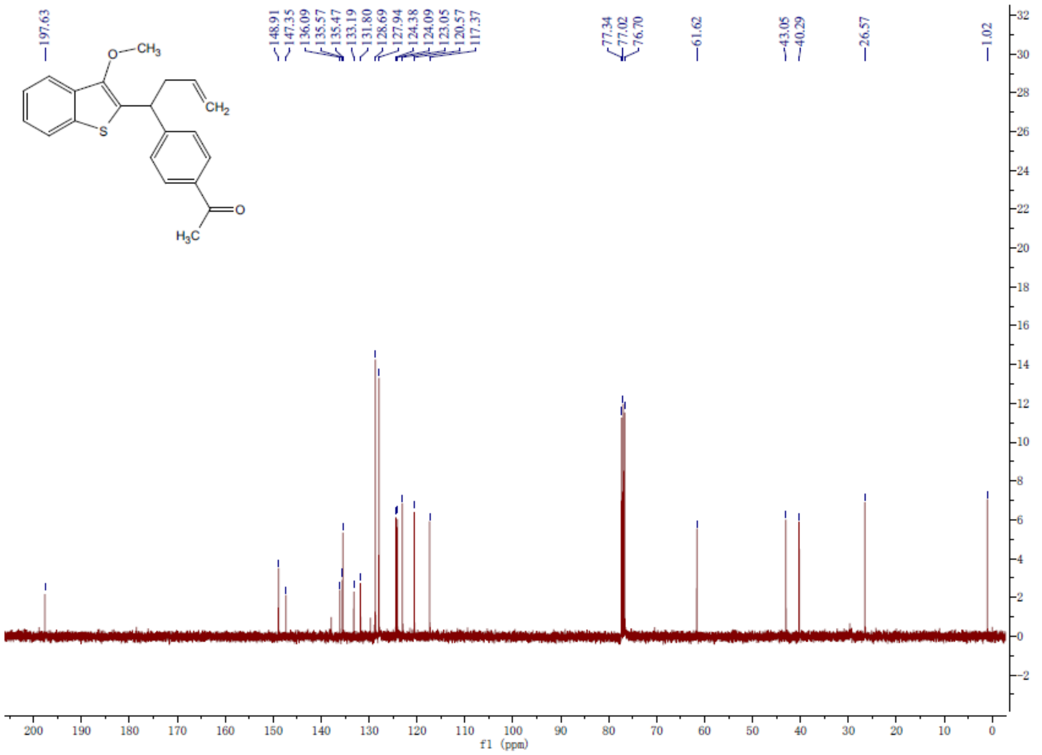
197.63
148.91 147.35 136.09 135.57 135.47 133.19 131.80 128.69 127.94 124.38 124.09 123.05 120.57 117.37
77.34 77.02 76.70
61.62
43.05 40.29
26.57
1.02
O
CH3
CH2
S
O
H3C
f1 (ppm)

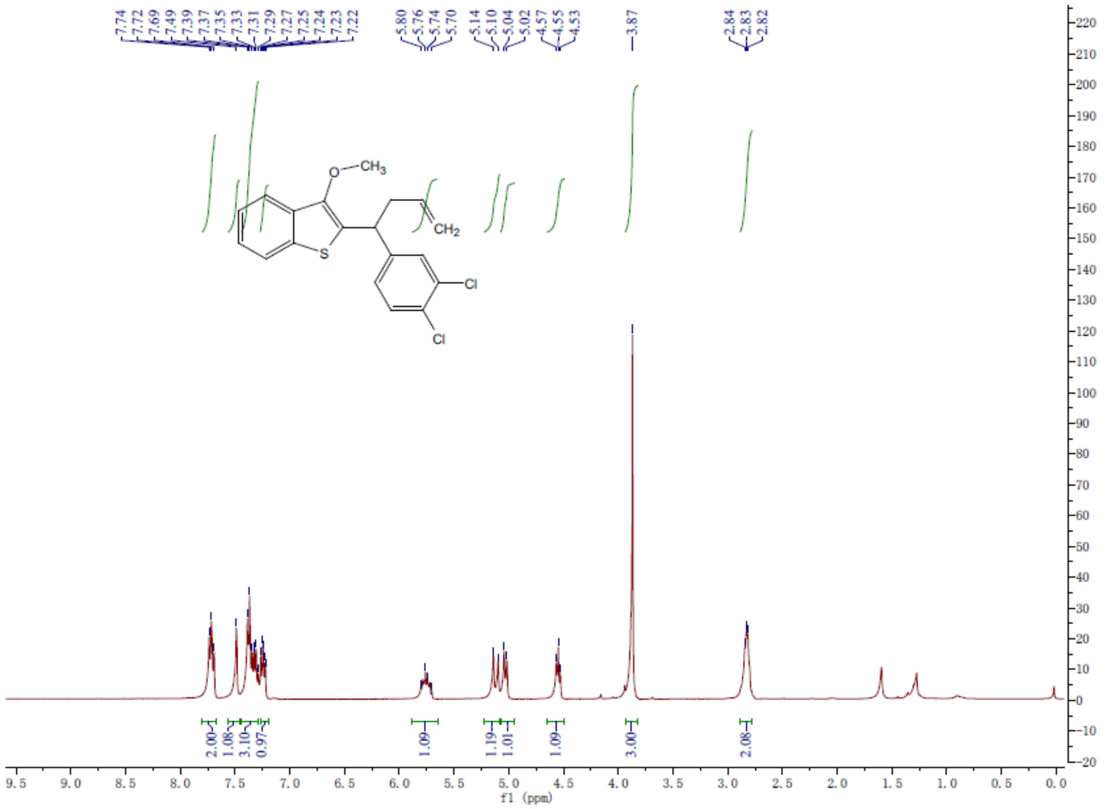
7.74 7.72 7.69 7.49 7.39 7.37 7.35 7.33 7.31 7.29 7.27 7.25 7.24 7.23 7.22
5.80 5.76 5.74 5.70
5.14 5.10 5.04 5.02
4.57 4.55 4.53
3.87
2.84 2.83 2.82
O
CH3
CH2
S
Cl
Cl
2.00 1.08 3.10 0.97
1.09
1.19 1.01
1.09
3.00
2.08
f1 (ppm)

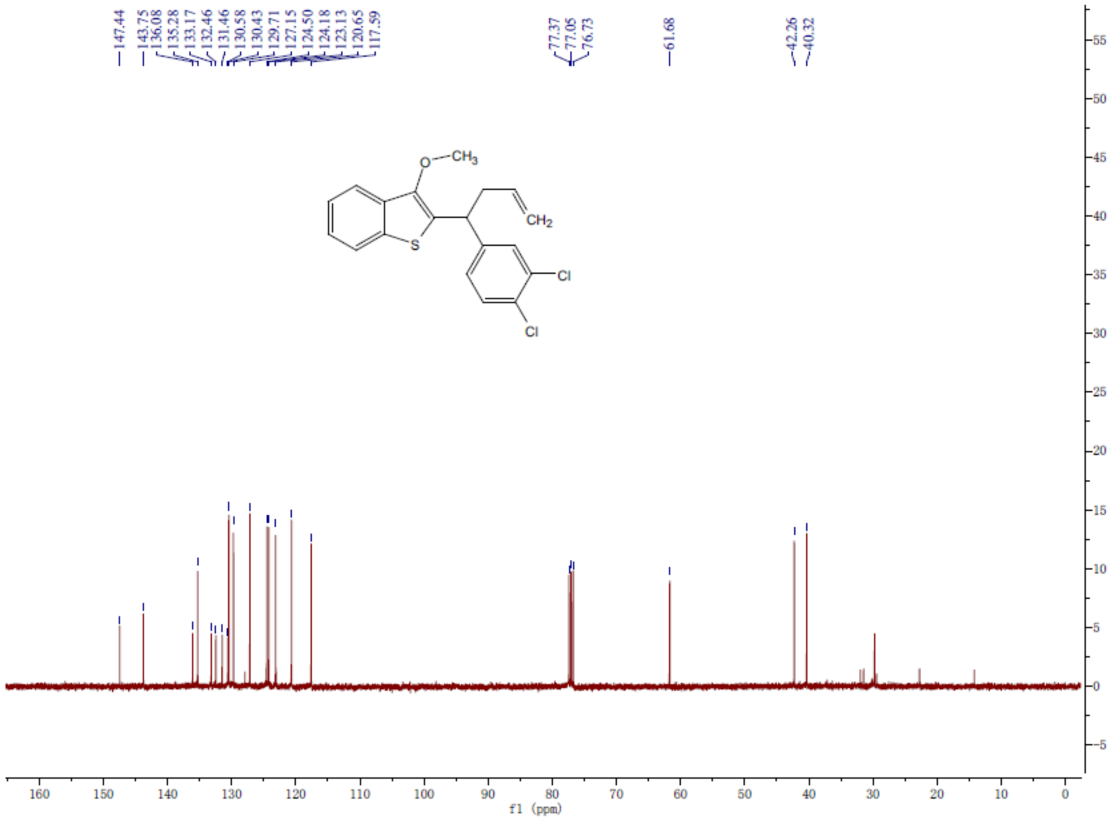
147.44 143.75 136.08 135.28 133.17 132.46 131.46 130.58 130.43 129.71 127.15 124.50 124.18 123.13 120.65 117.59
77.37 77.05 76.73
61.68
42.26 40.32
O
CH3
CH2
S
Cl
Cl
f1 (ppm)

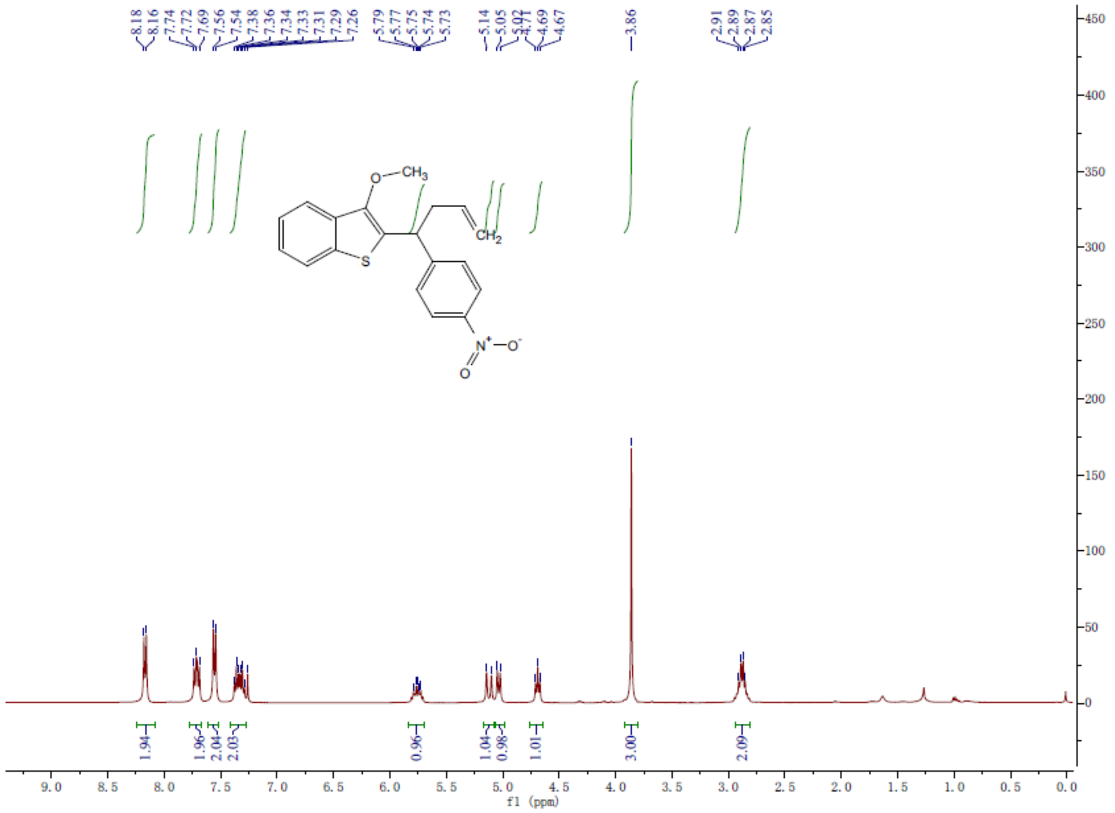
8.18
8.16
7.74
7.72
7.69
7.56
7.54
7.38
7.36
7.34
7.33
7.31
7.29
7.26
5.79
5.77
5.75
5.74
5.73
5.14
5.05
5.02
4.71
4.69
4.67
3.86
2.91
2.89
2.87
2.85
O—CH3
CH2
S
N+—O-
O
1.94
1.96
2.04
2.03
0.96
1.04
0.98
1.01
3.00
2.09
f1 (ppm)

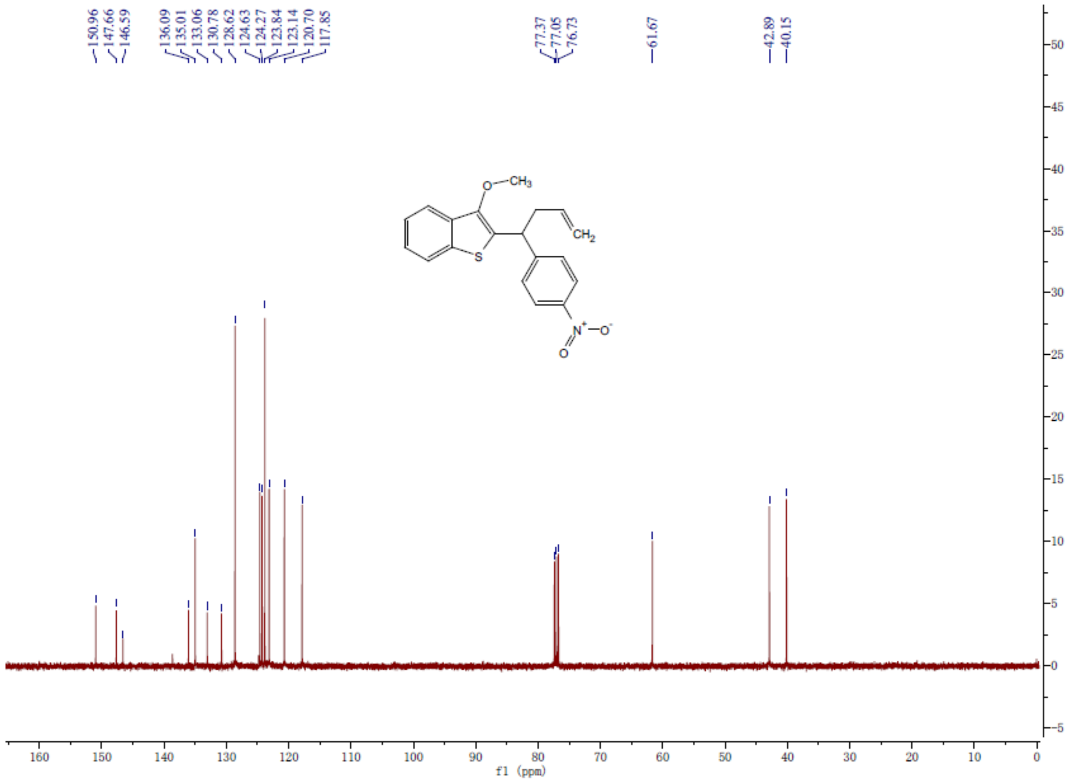
150.96
147.66
146.59
136.09
135.01
133.06
130.78
128.62
124.63
124.27
123.84
123.14
120.70
117.85
77.37
77.05
76.73
61.67
42.89
40.15
O—CH3
CH2
S
N+—O-
O
f1 (ppm)

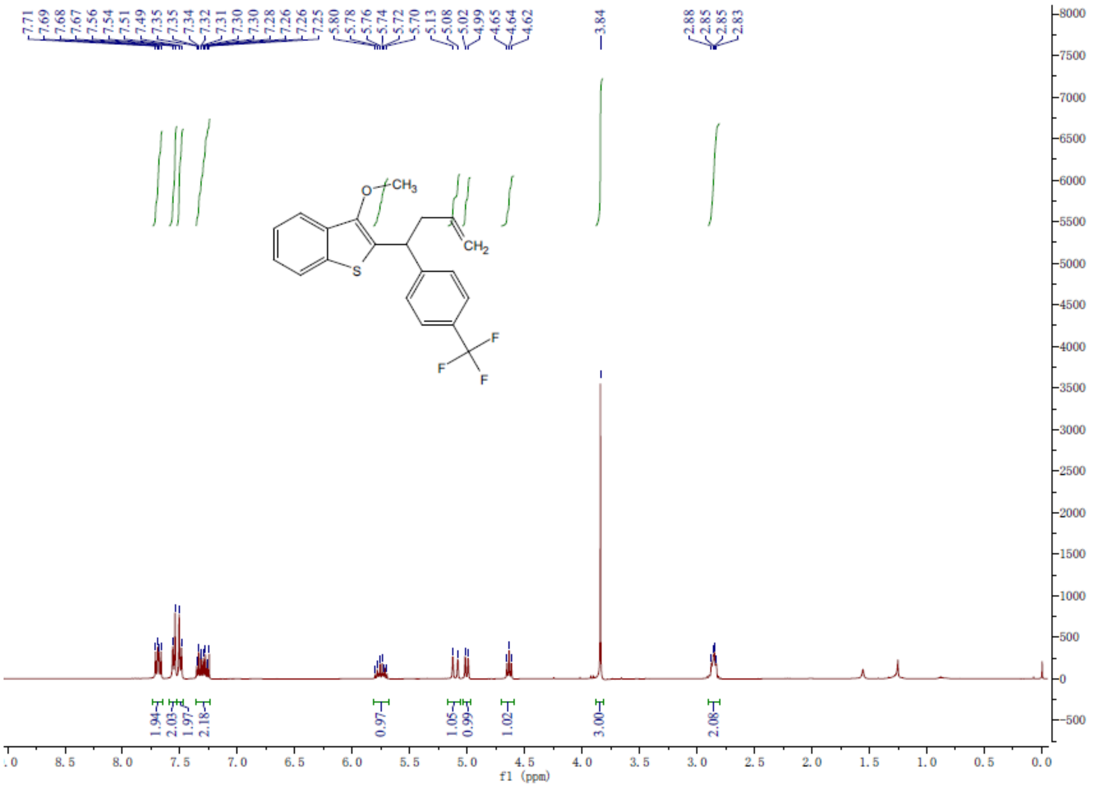

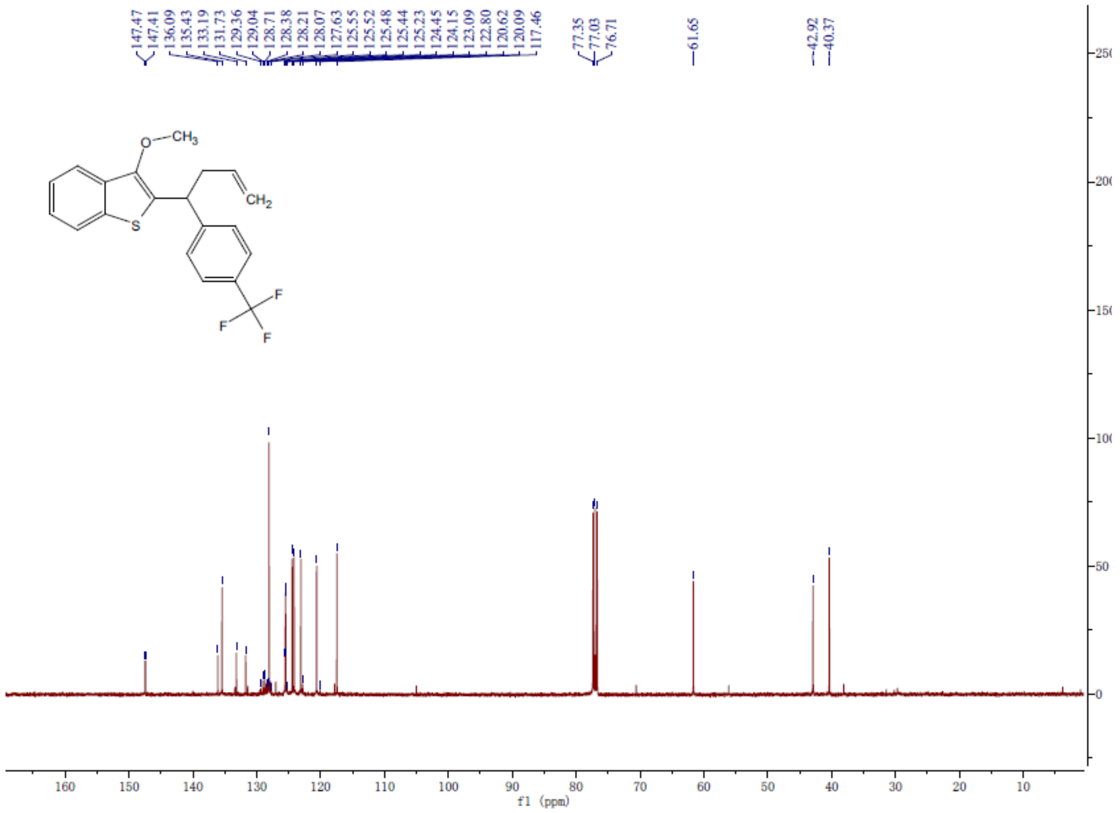

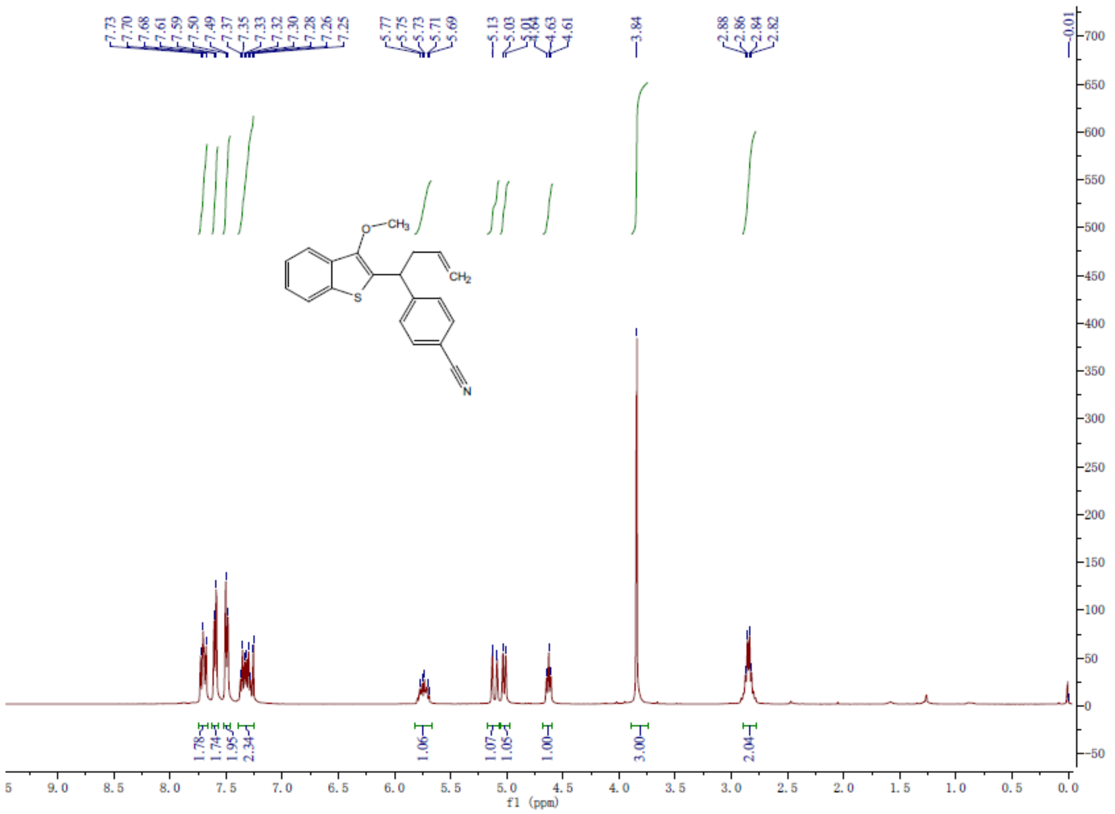

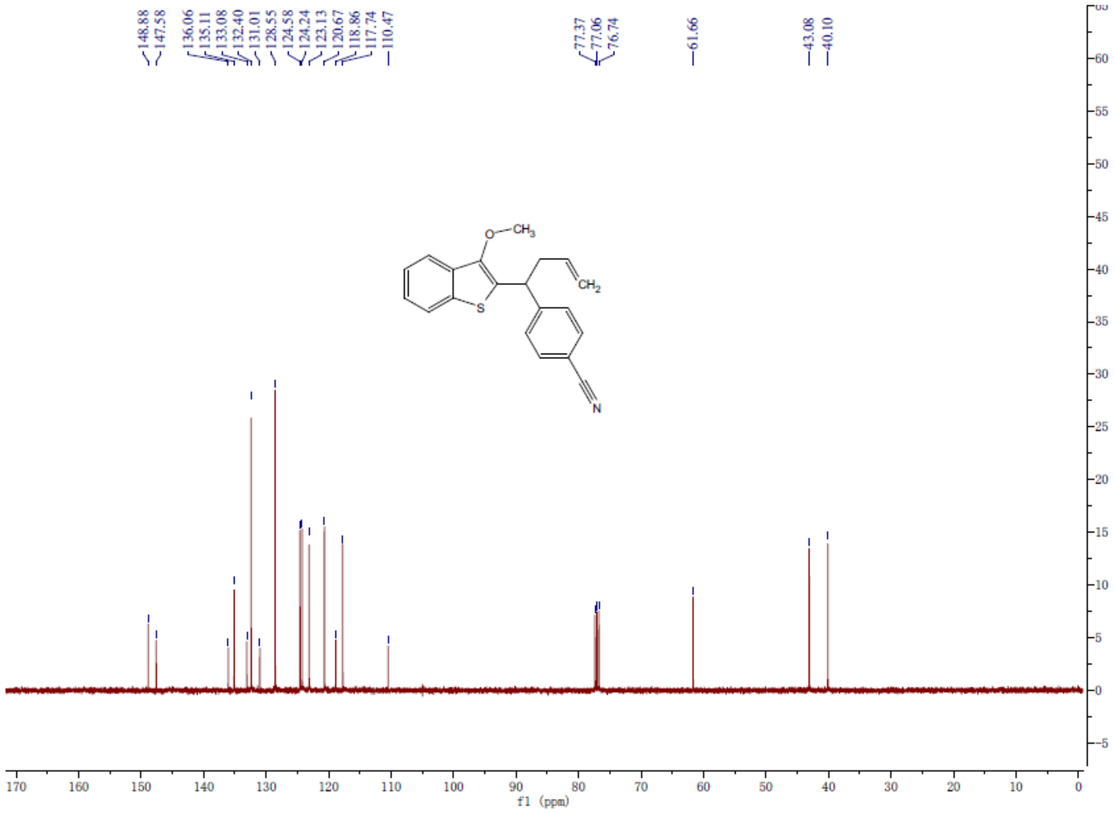

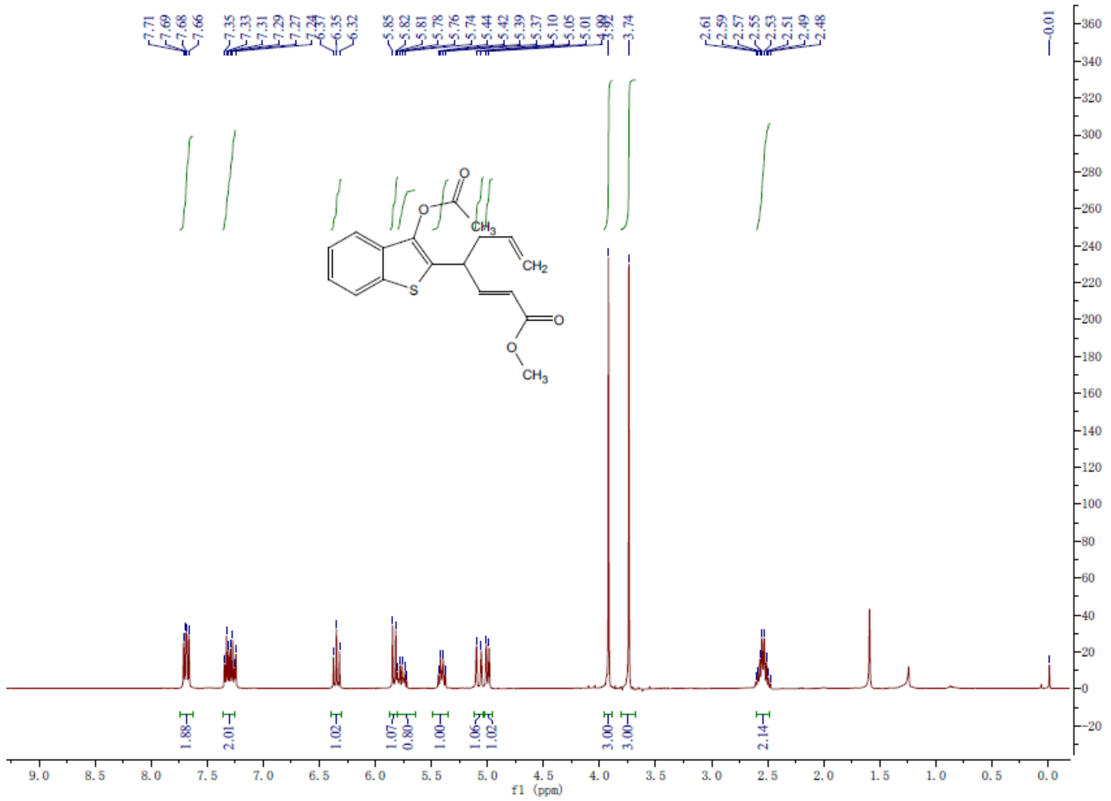

7.71
7.69
7.68
7.66
7.35
7.33
7.31
7.29
7.27
7.24
6.37
6.35
6.32
5.85
5.82
5.81
5.78
5.76
5.74
5.44
5.42
5.39
5.37
5.10
5.05
5.01
3.92
3.74
2.61
2.59
2.57
2.55
2.53
2.51
2.49
2.48
-0.01
1.88
2.01
1.02
1.07
0.80
1.00
1.06
1.02
3.00
3.00
2.14
CH3
CH2
S
O
CH3
f1 (ppm)

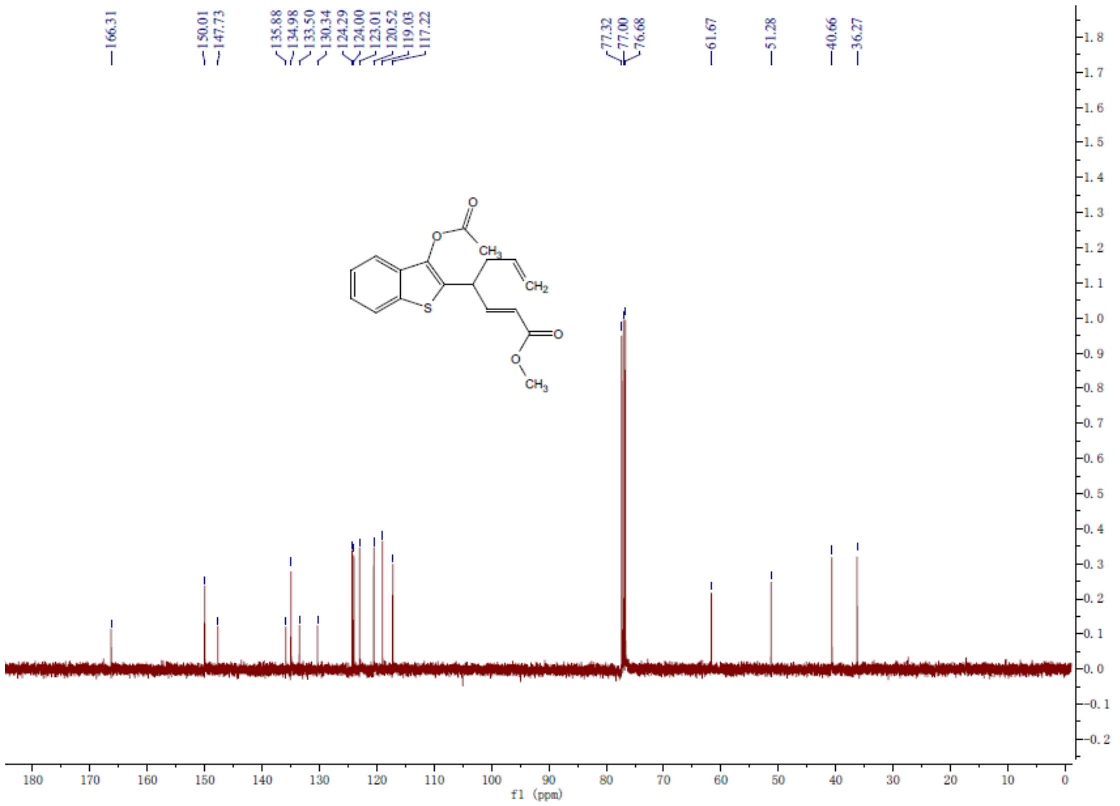

166.31
150.01
147.73
135.88
134.98
133.50
130.34
124.29
124.00
123.01
120.52
119.03
117.22
77.32
77.00
76.68
61.67
51.28
40.66
36.27
CH3
CH2
S
O
CH3
f1 (ppm)

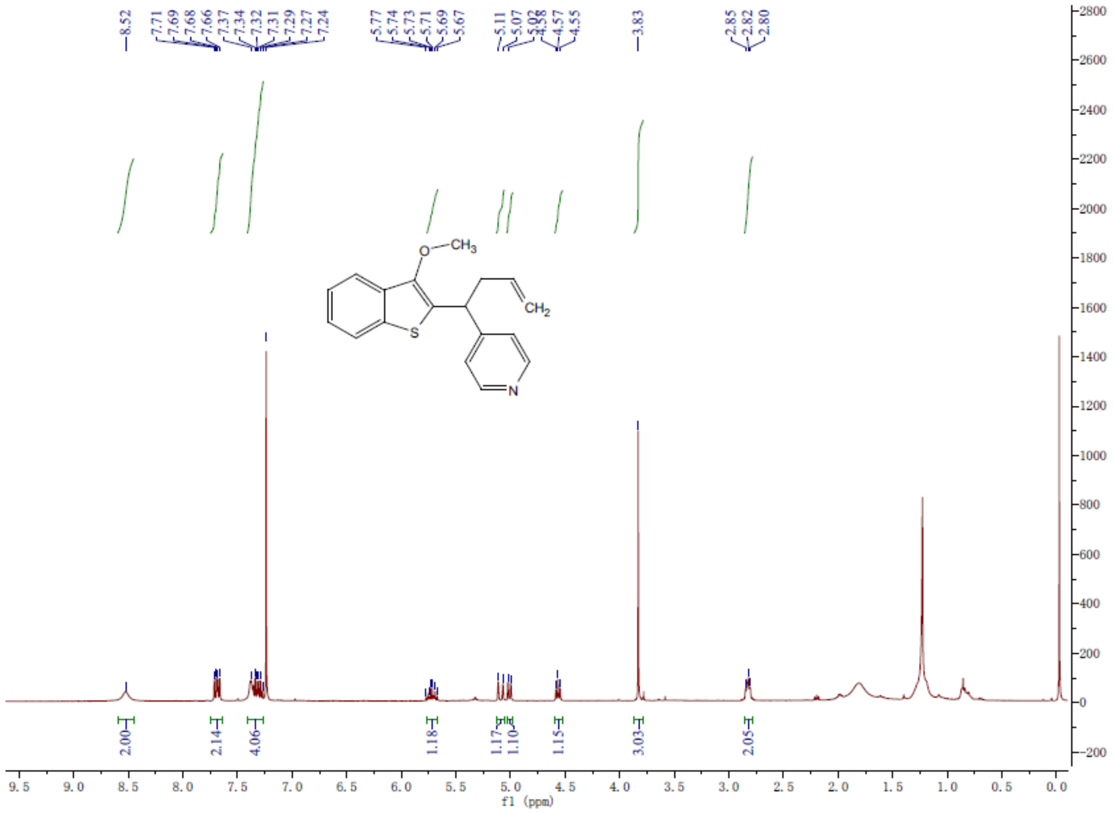

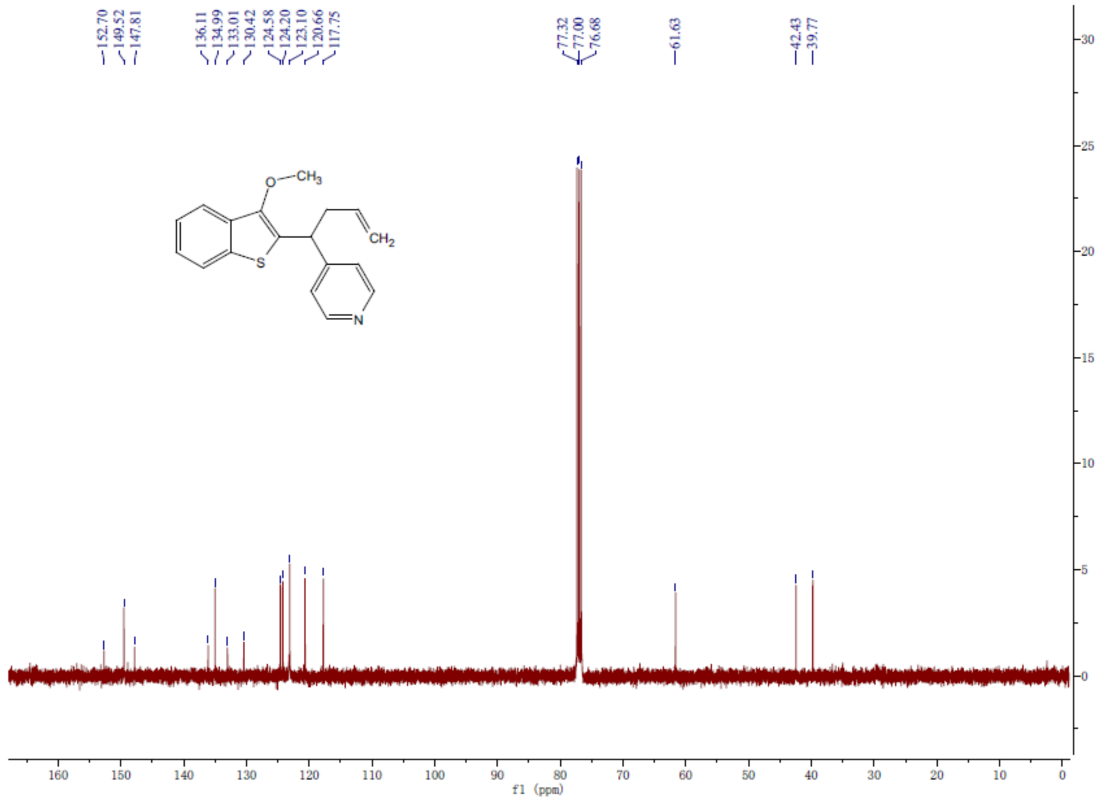

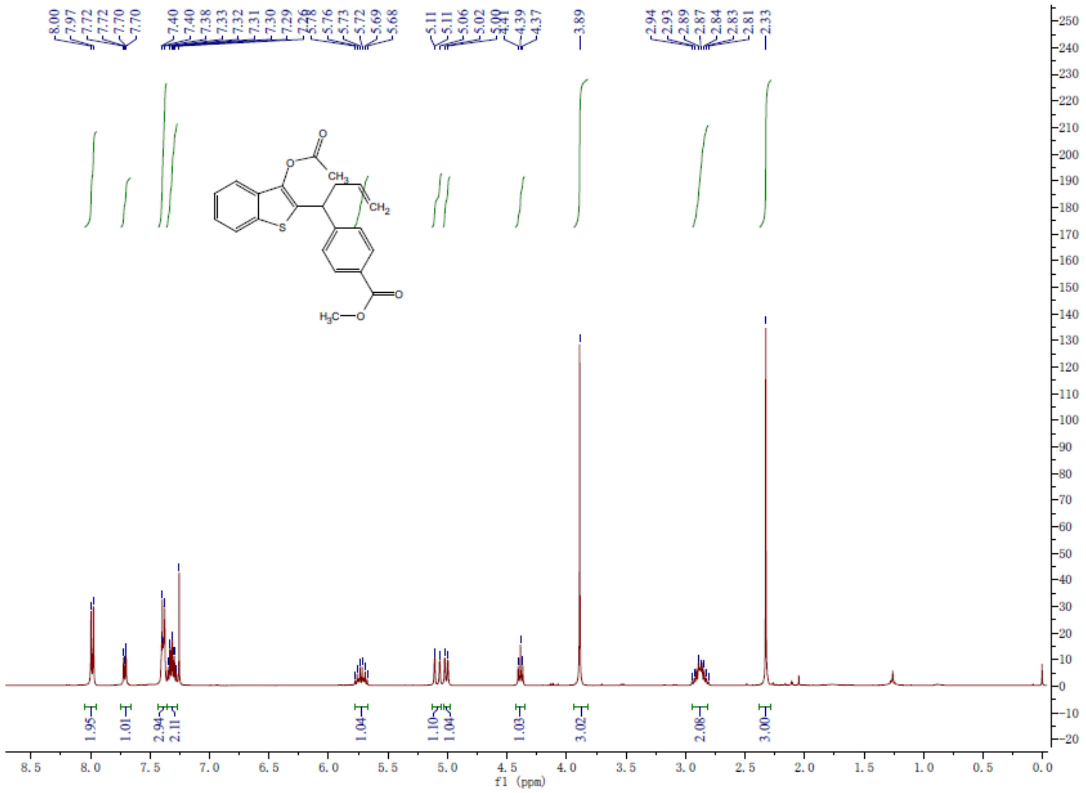
f1 (ppm)

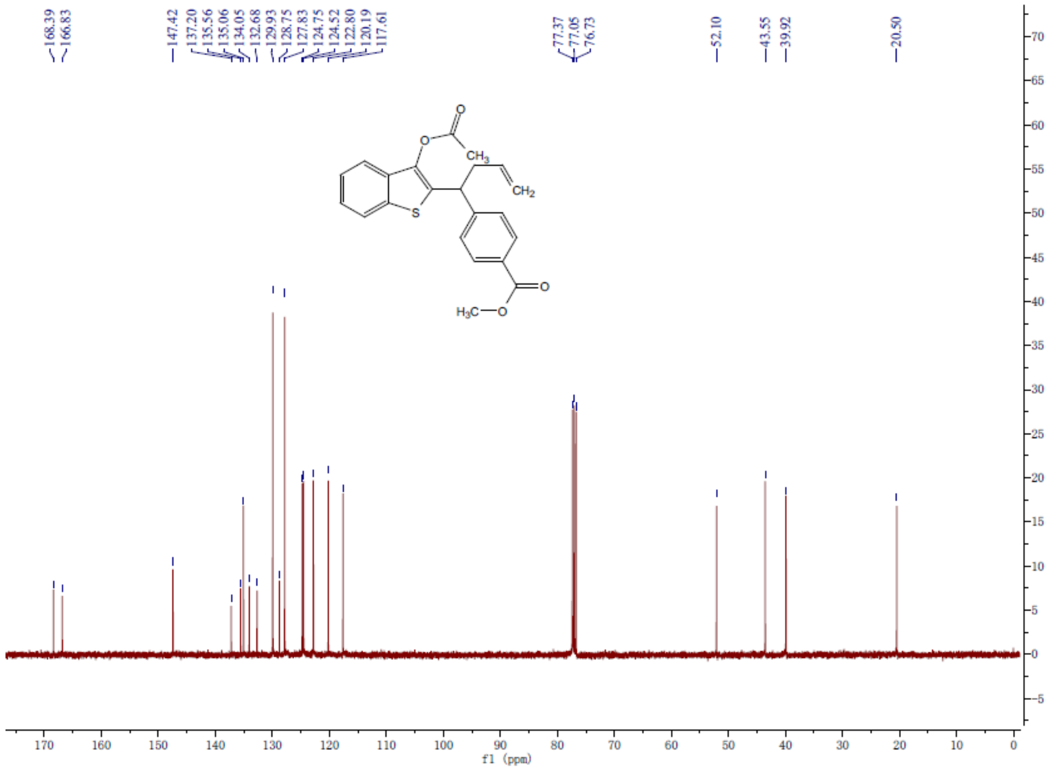
f1 (ppm)

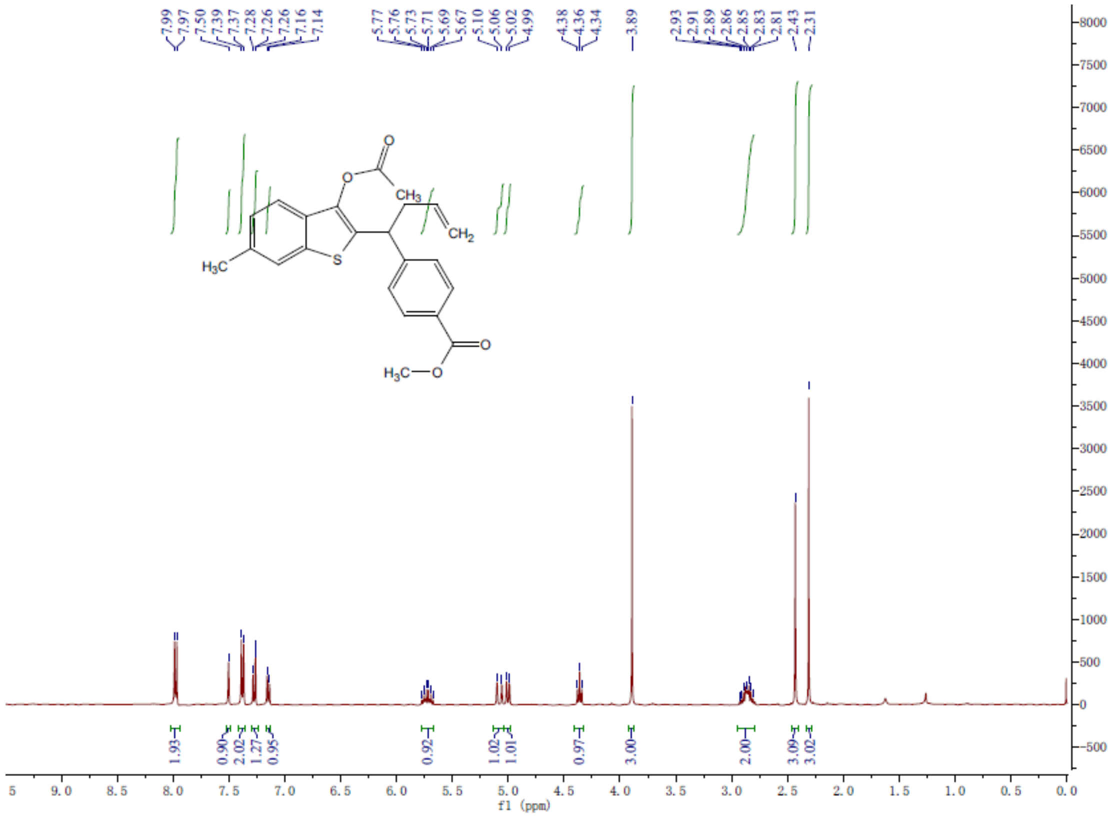

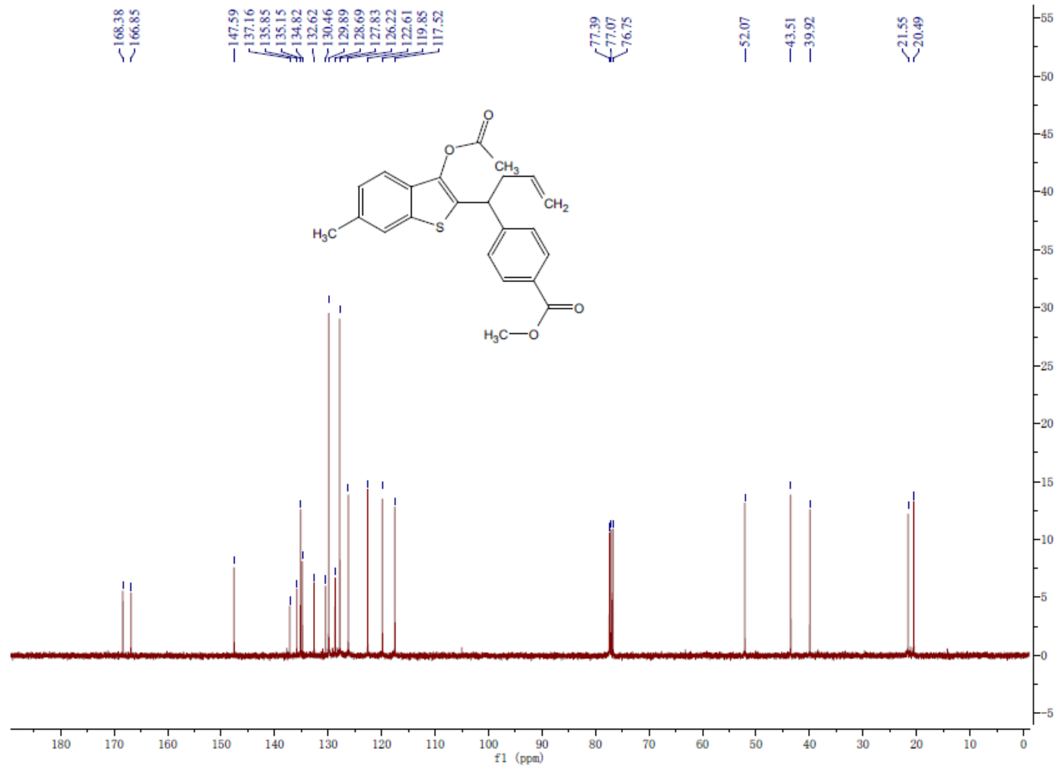

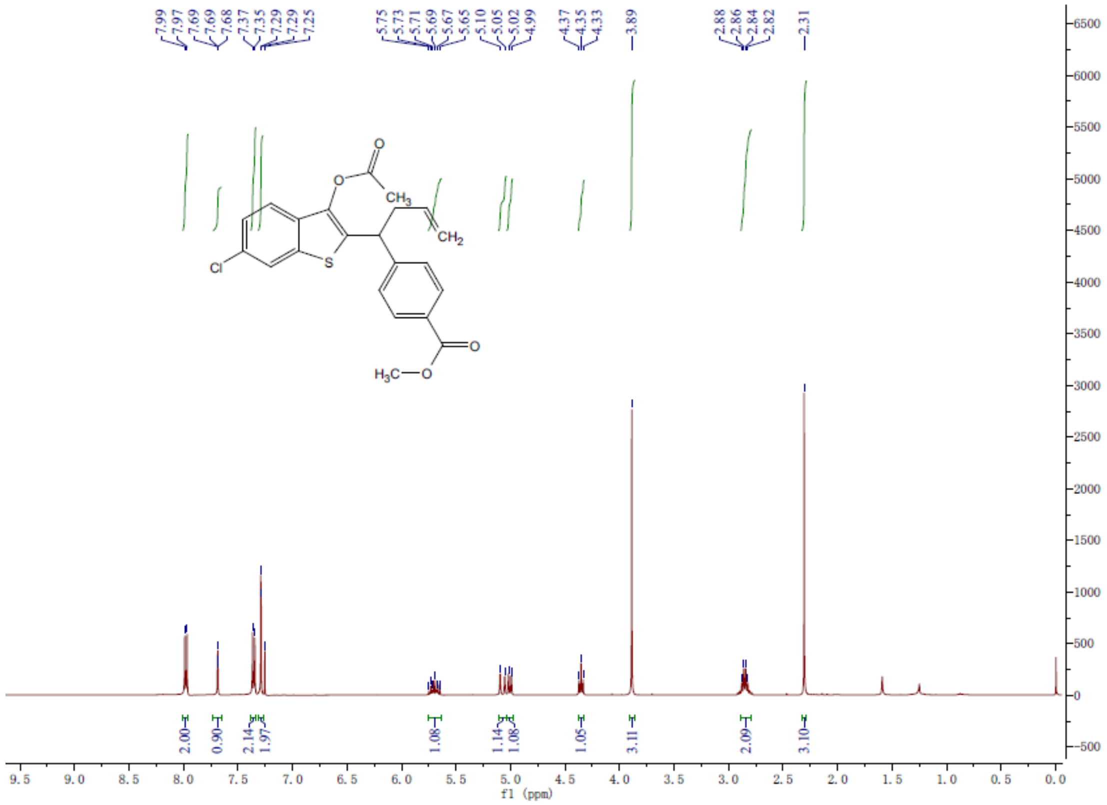
7.99 7.97 7.69 7.69 7.68 7.37 7.35 7.29 7.29 7.25
5.75 5.73 5.71 5.69 5.67 5.65 5.10 5.05 5.02 4.99
4.37 4.35 4.33
3.89
2.88 2.86 2.84 2.82
2.31
2.00 0.90 2.14 1.97 1.08 1.14 1.08 1.05 3.11 2.09 3.10
Cl
S
O
CH3
CH2
H3C
f1 (ppm)

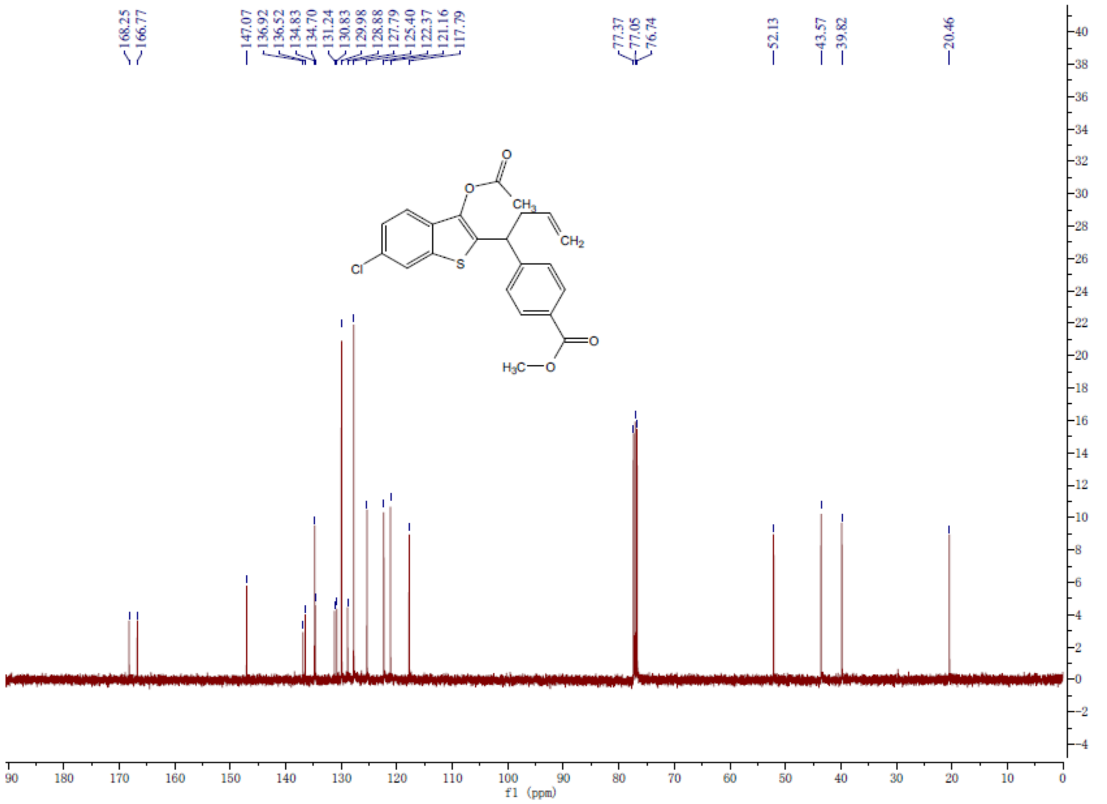
168.25 166.77
147.07 136.92 136.52 134.83 134.70 131.24 130.83 129.98 128.88 127.79 125.40 122.37 121.16 117.79
77.37 77.05 76.74
52.13
43.57 39.82
20.46
Cl
S
O
CH3
CH2
H3C
f1 (ppm)

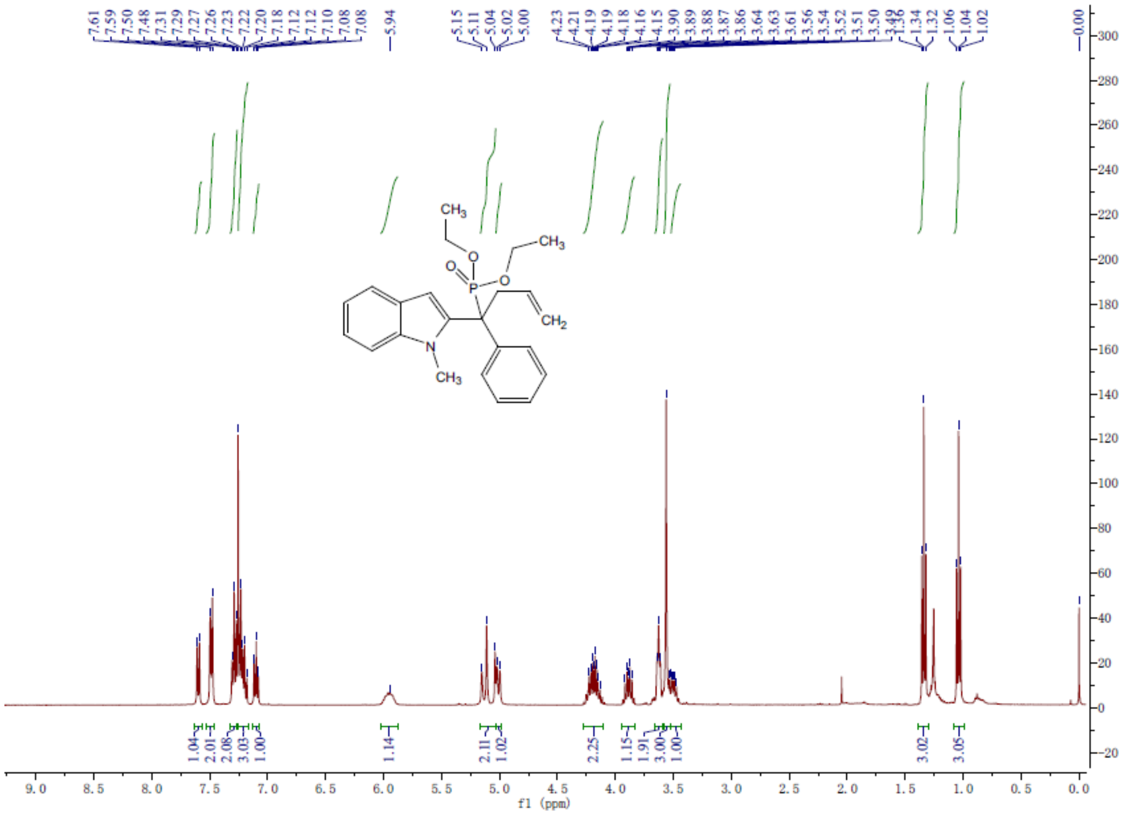
7.61 7.59 7.50 7.48 7.31 7.29 7.27 7.26 7.23 7.22 7.20 7.18 7.12 7.12 7.10 7.08 7.08
5.94
5.15 5.11 5.04 5.02 5.00
4.23 4.21 4.19 4.19 4.18 4.16 4.15 3.90 3.89 3.88 3.87 3.86 3.64 3.63 3.61 3.56 3.54 3.52 3.51 3.50 3.49 1.36 1.34 1.32 1.06 1.04 1.02
0.00
CH3
O
O
P
CH3
CH2
N
CH3
1.04 2.01 2.08 3.03 1.00 1.14 2.11 1.02 2.25 1.15 1.91 3.00 1.00 3.02 3.05
f1 (ppm)

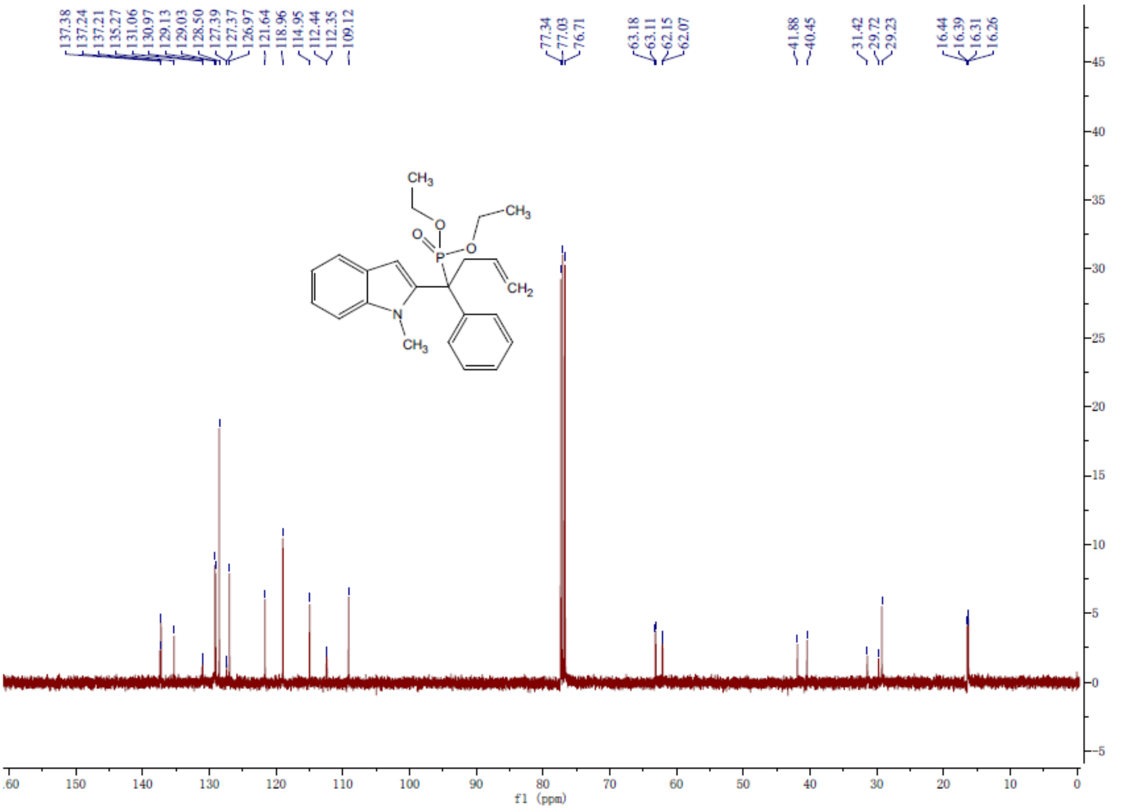
137.38 137.24 137.21 135.27 131.06 130.97 129.13 129.03 128.50 127.39 127.37 126.97 121.64 118.96 114.95 112.44 112.35 109.12
77.34 77.03 76.71
63.18 63.11 62.15 62.07
41.88 40.45
31.42 29.72 29.23
16.44 16.39 16.31 16.26
CH3
O
O
P
CH3
CH2
N
CH3
f1 (ppm)

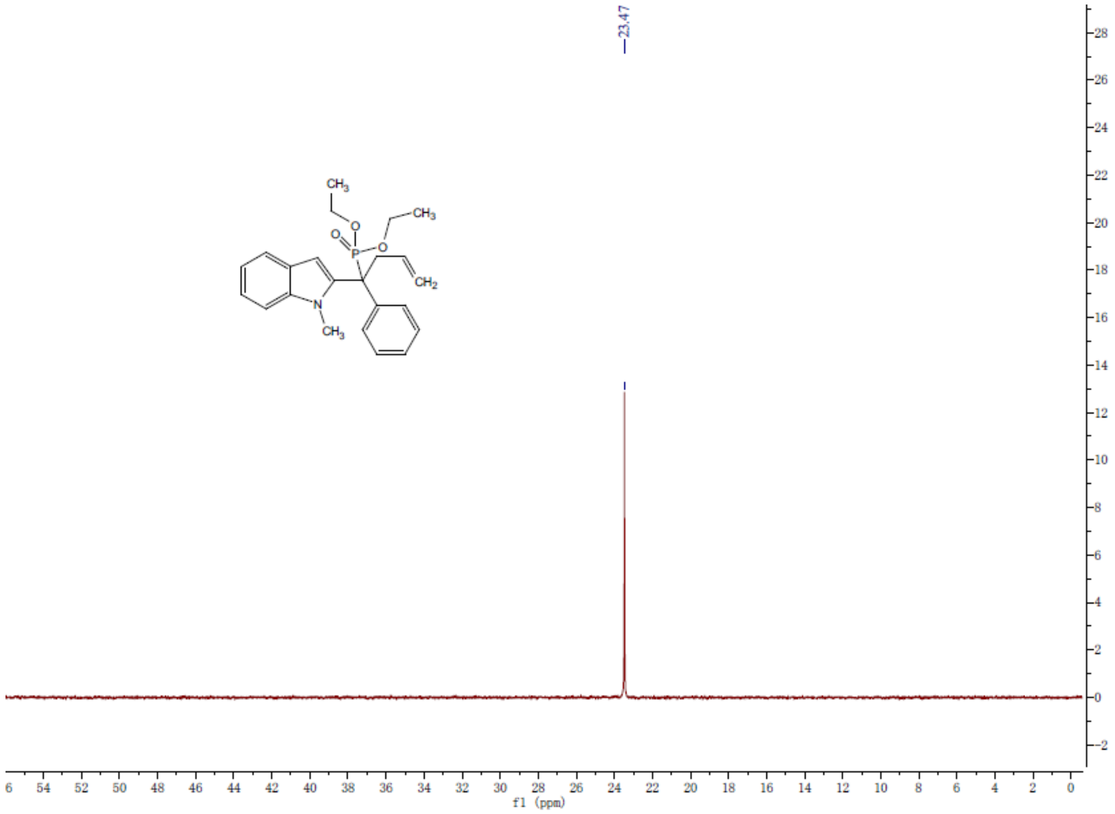

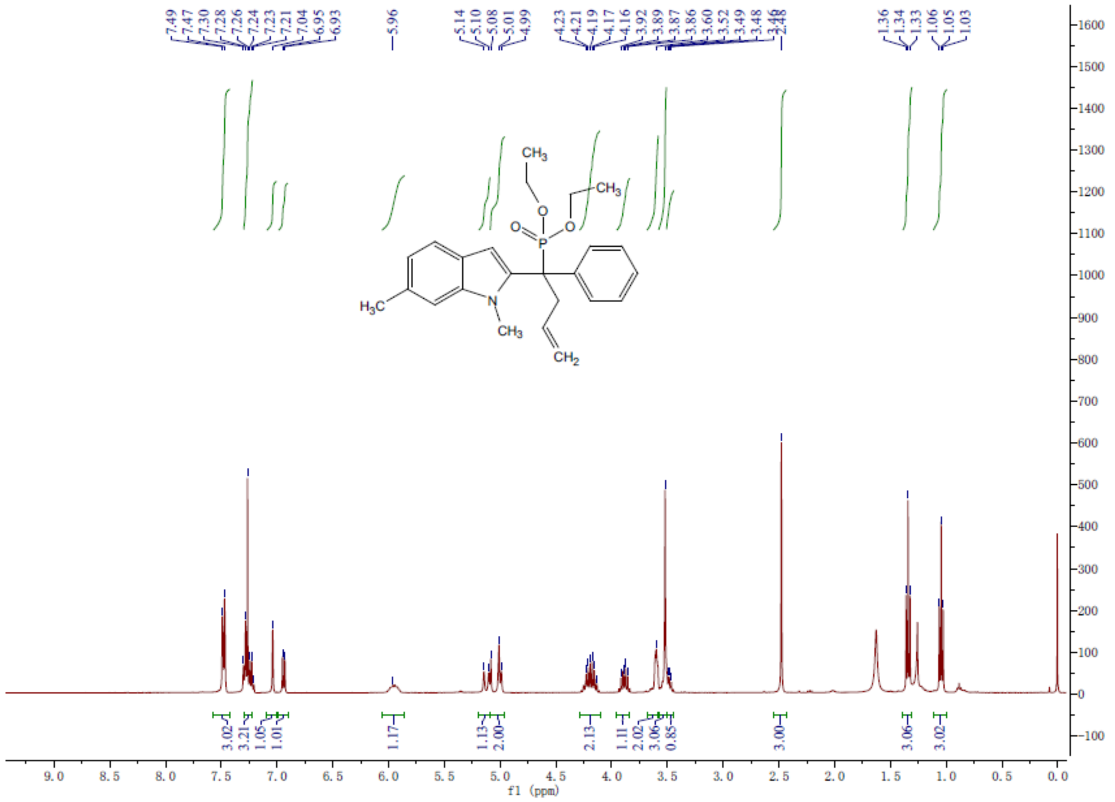

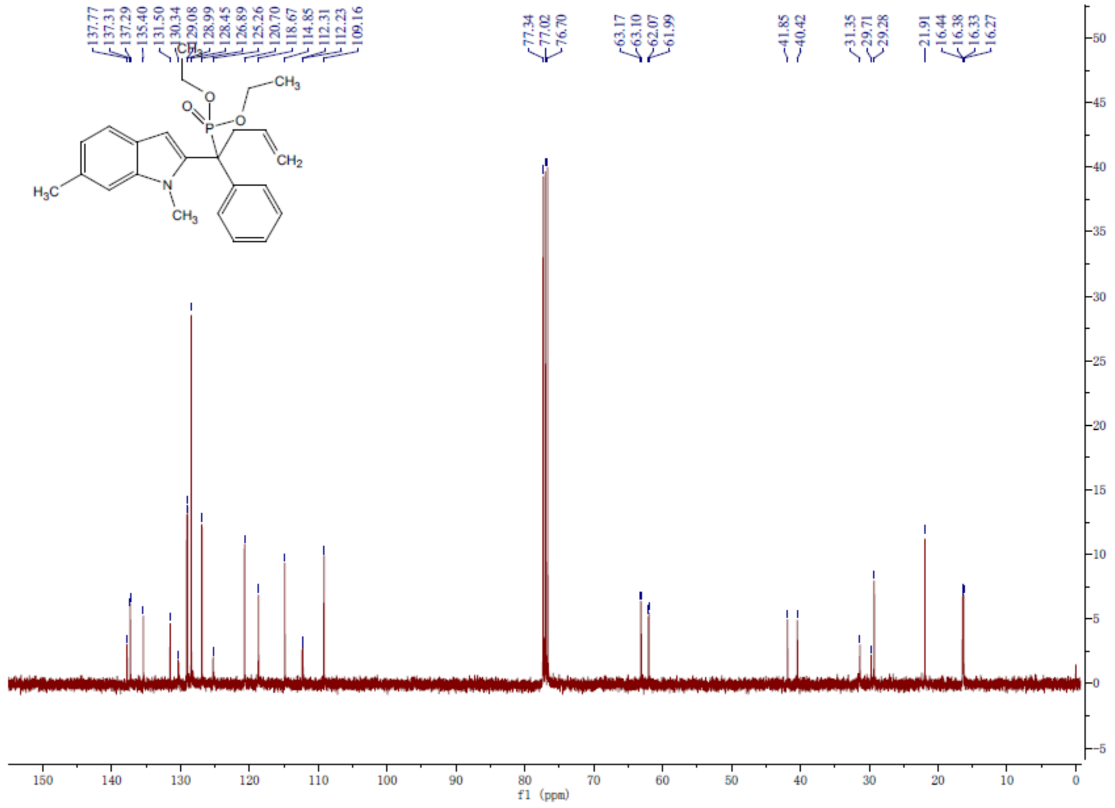
137.77
137.31
137.29
135.40
131.50
130.34
129.08
128.99
128.45
126.89
125.26
120.70
118.67
114.85
112.31
112.23
109.16
77.34
77.02
76.70
63.17
63.10
62.07
61.99
41.85
40.42
31.35
29.71
29.28
21.91
16.44
16.38
16.33
16.27
f1 (ppm)

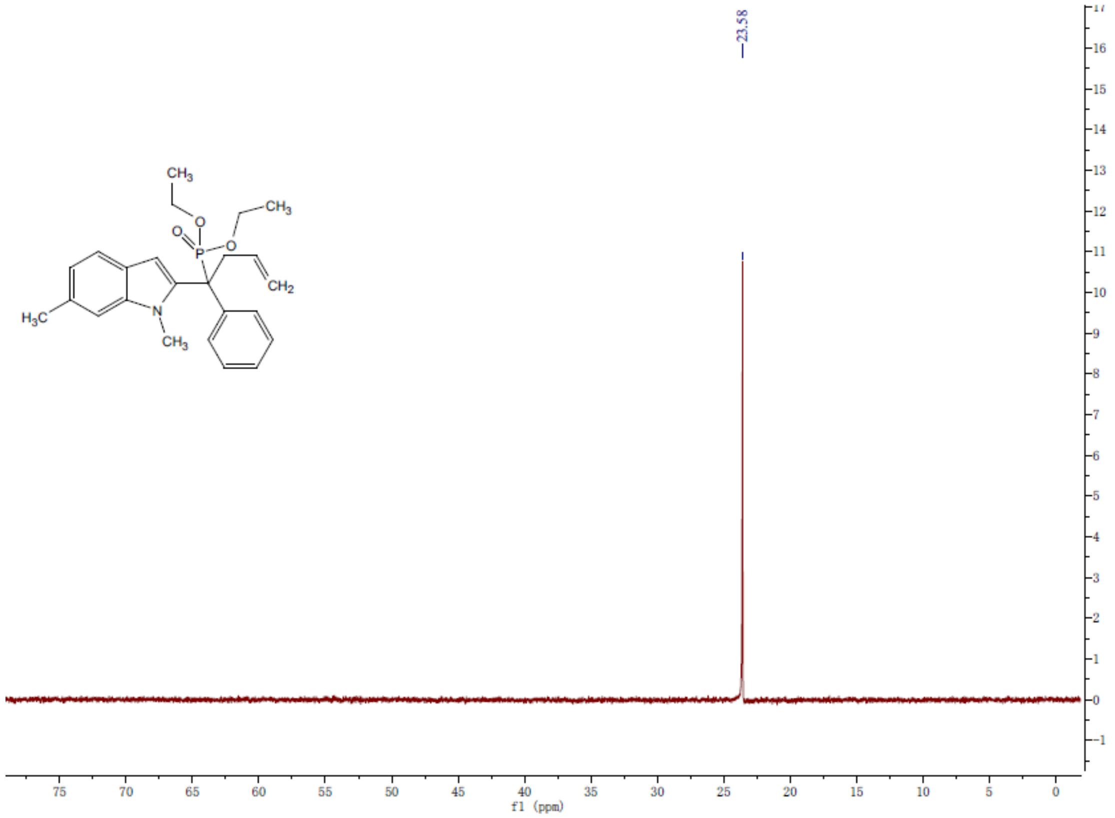
23.58
f1 (ppm)

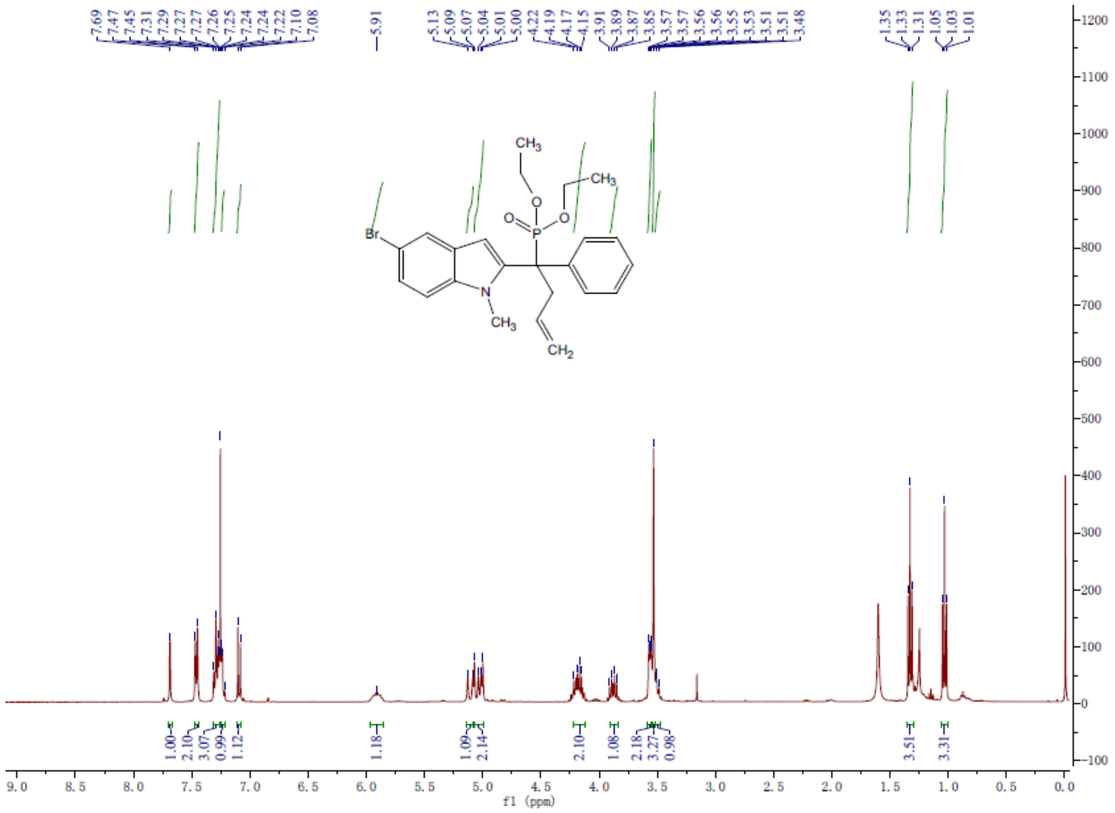

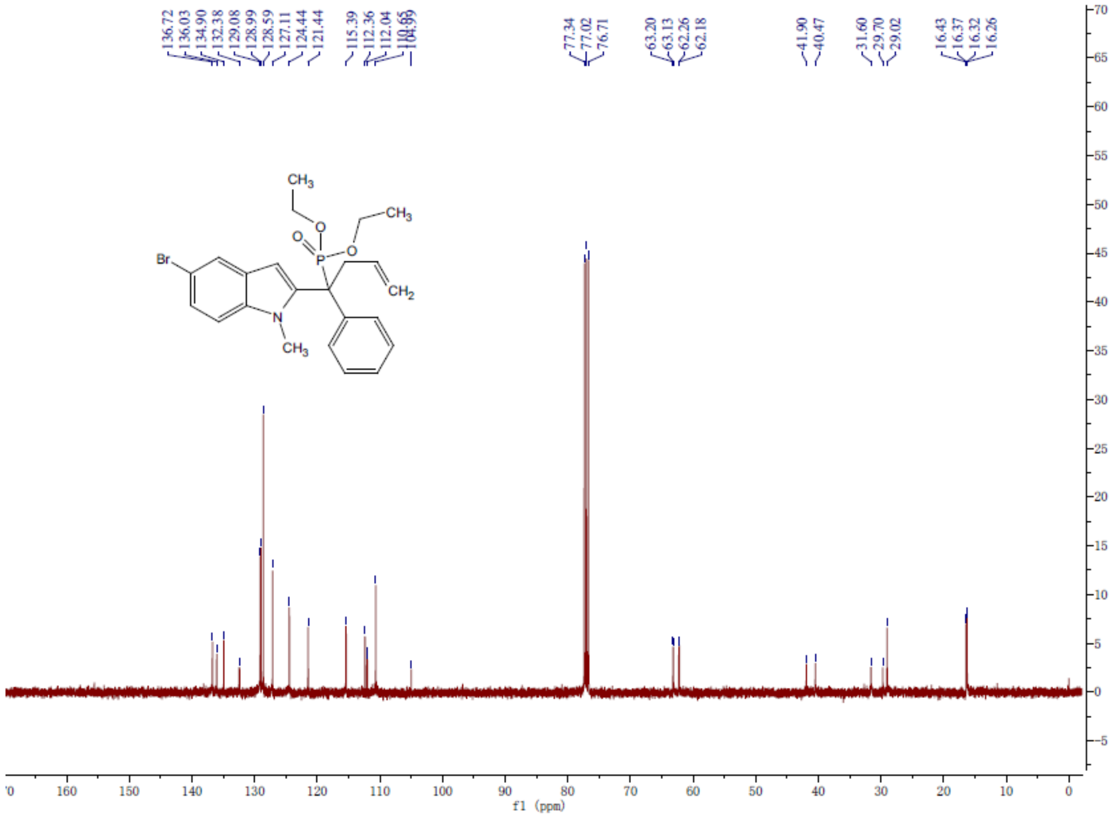

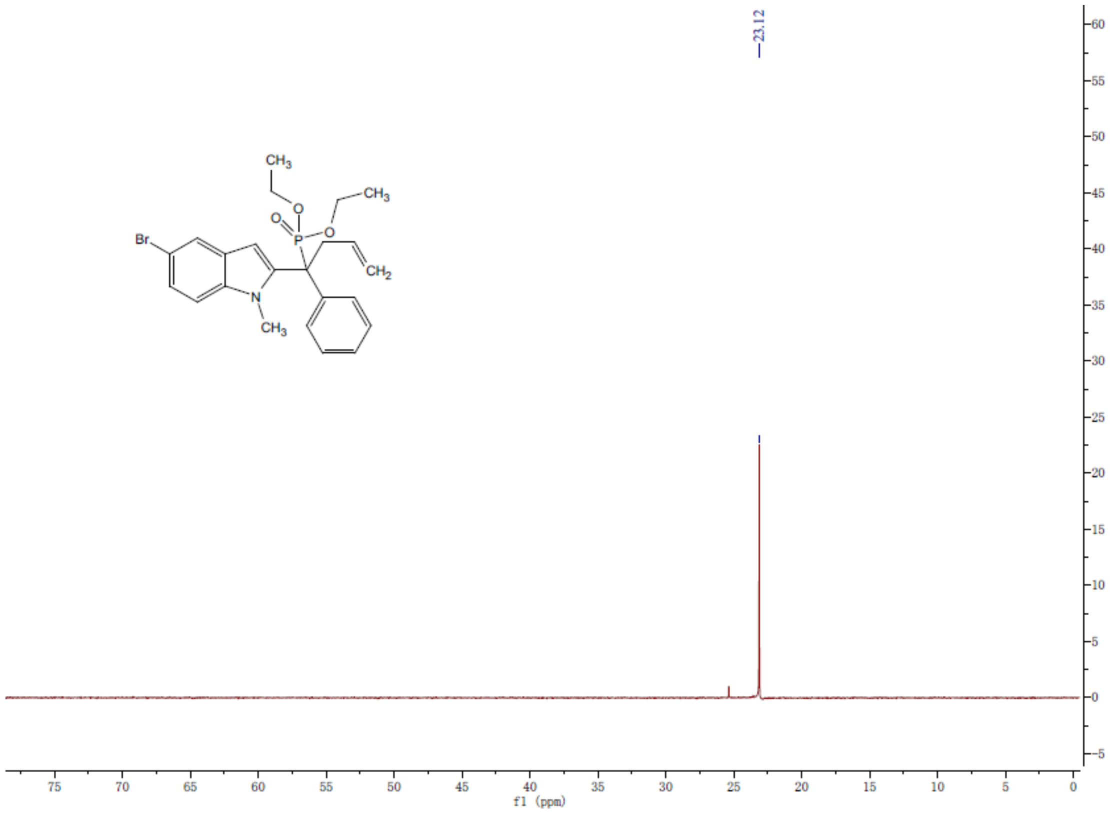

23.12
f1 (ppm)
